# Synthetic Lethal Therapy Based on Dimorphism for Systemic Infection of Drug‐Resistant *Candida albicans*

**DOI:** 10.1002/advs.202518196

**Published:** 2025-12-03

**Authors:** Yang Gao, Jiahe Su, Jiawei Yuan, Qinyan Cao, Yue Wu, Yuyang Xiao, Guang Yang, Ruomu Xia, Jingpeng Yang, Yanan Li, Lina Wu, He Huang, Lingtong Meng

**Affiliations:** ^1^ State Key Laboratory of Microbial Technology School of Food and Pharmaceutical Engineering Nanjing Normal University Nanjing 210023 China; ^2^ Shanghai Institute for Advanced Immunochemical Studies ShanghaiTech University Shanghai 201210 China; ^3^ College of Life Sciences Beijing Normal University Beijing 100091 China

**Keywords:** Candida albicans, dimorphism, hyphae, rapamycin, synthetic lethal therapies

## Abstract

Systemic infections of *Candida albicans* are dire, with a mortality rate reaching 60%. The transformation between the yeast and hyphae states of *C. albicans* resists existing medications and the body's immune defenses. Some currently available drugs only inhibit the growth of the yeast‐form of *C. albicans*. Therefore, a synthetic lethal therapy that leverages the dimorphism of *C. albicans* is developed. This therapy utilizes rapamycin to eliminate yeast‐like *C. albicans* through the mammalian target of rapamycin pathway. Simultaneously, deferasirox restricts iron resources, thereby inhibiting polarization and hyphae formation. As a result, *C. albicans* cannot evade the lethal effects of drugs and immune cells by undergoing morphological transformations. Furthermore, inspired by the recognition process of fungi by macrophages, macrophage membranes with high expression of dectin‐1 are utilized to deliver rapamycin and deferasirox. The synthetic lethal therapy based on dimorphism significantly improves the survival rate of mice with systemic infections, thereby offering a promising strategy for treating drug‐resistant *C. albicans* infections.

## Graphical abstract

To tackle the alarming mortality rate linked to *Candida albicans* infections, a synthetic lethal strategy precisely aimed at the two distinct forms of this fungus: yeast and hyphae is formulated. Ultimately, through the innovative use of macrophage membranes for drug delivery, the effectiveness of this strategy is substantially boosted while simultaneously reducing the immunological side effects associated with the medication.

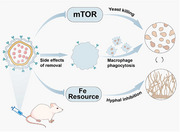

## 1 Introduction

*Candida albicans (C. albicans)* is the most common fungal species in human pathogenic microorganisms. *C. albicans* poses a significant global health threat as one of the most critical opportunistic fungal pathogens. Systemic infections with this fungus primarily affect immunocompromised individuals, including patients with cancer, AIDS, and COVID‐19. In the past few decades, there have been more than 600 000 cases of candida bloodstream infection worldwide every year, and the mortality rate has exceeded 60%.^[^ ^1^, ^2^, ^3^ ^]^ However, the prevalent antifungal drugs commonly exhibit resistance, with the clinical drug fluconazole (FLU) demonstrating particularly severe drug resistance issues. Even newly emerging drugs, such as echinocandins, have reportedly begun to show signs of resistance.^[^ ^4^ ^]^ A primary factor contributing to drug resistance is the alteration of drug targets within cells, rendering the drugs ineffective.^[^ ^5^ ^]^ To address the issue of *C. albicans* drug resistance and enhance therapeutic outcomes, we propose identifying a drug possessing non‐traditional antifungal target.

Mammalian target of rapamycin (mTOR) plays a crucial role in regulating the metabolism and growth of numerous cell types. Notably, rapamycin (RAP), a potent inhibitor of mTOR, has demonstrated remarkable inhibitory effects on the progression of *C. albicans*.^[^ ^1^, ^6^ ^]^ The mechanism of action of RAP on *C. albicans* has been extensively reported. Upon entering the cell, RAP binds to the immunophilin FKBP12 to form a complex. This complex then binds to the FRB (FKBP12‐rapamycin binding) domain of the Tor1 protein kinase in the mTOR pathway, thereby inhibiting the kinase activity of Tor1 and interfering with multiple biological processes associated with the growth of *C. albicans*.^[^ ^7^, ^8^ ^]^ Despite this, RAP has yet to be harnessed for antifungal therapy. Our previous work revealed that RAP effectively inhibits the growth of *C. albicans* but paradoxically promotes hyphae development. *C. albicans*' defining characteristic distinguishing it from ordinary fungi is its dimorphism, enabling it to transition between yeast and hyphae forms. These two forms have distinct functions. In the bloodstream, *C. albicans* adopts a yeast form, which facilitates its dissemination and reproduction. Conversely, when it needs to invade internal organs, it transforms into hyphae to enhance adherence and invasion, while discharging numerous virulence factors.^[^ ^9^, ^10^ ^]^ The transition between hyphae and yeast morphologies is crucial for *C. albicans* to achieve systemic invasion. Furthermore, this biphasic transformation also serves as a defense mechanism against existing antifungal drugs.^[^ ^11^ ^]^ However, some current antifungal drugs are solely efficacious against yeast‐form fungi. For instance, FLU fails to fully address the therapeutic challenges associated with the dimorphism of *C. albicans*.^[^ ^12^, ^13^ ^]^ Therefore, effectively inhibiting hyphae formation is critical in overcoming fungal drug resistance and improving the prognosis of systemic *C. albicans* infection.

**Figure 1:**
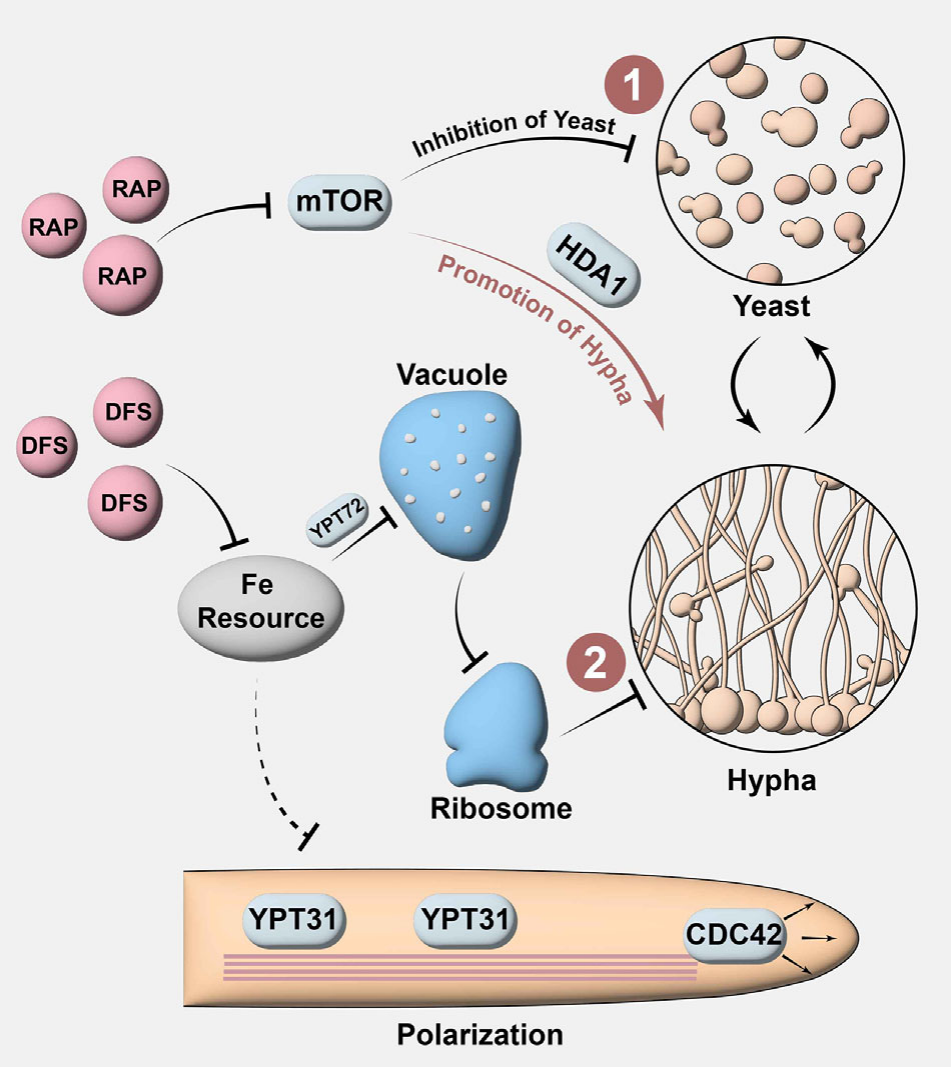
Schematic diagram of synthetic lethal therapy involving RAP and DFS. RAP effectively halts the growth and metabolism of *C. albicans* by suppressing mTOR, ultimately leading to the death of the yeast. Concurrently, *HDA1* activation fosters hyphae development. Meanwhile, DFS restricts the cellular iron supply, inhibiting the transcription of *YPT72*, and causing vacuole disruption. This disruption triggers amino acid metabolism disorders and further compromises the function and quantity of ribosomes, resulting in an inability to produce functional proteins necessary for hyphae development and thus inhibiting hyphae growth. Additionally, DFS inhibits the vesicle transport pathway, which is responsible for hyphae polarization, further hindering hyphae development. The suppression of the two‐phase morphology of *C. albicans* prevents it from evading the lethal effects of drugs and immune cells through morphological transformation.

Iron ion is an indispensable trace element in the growth of fungi and has the highest content in the human body.^[^ ^14^ ^]^ In our previous work, we discovered that iron ions stimulate the growth of *C. albicans* hyphae. Based on this finding, we hypothesize that iron chelating agents may inhibit hyphae growth, thereby addressing the issue of RAP promoting hyphae development. Given its potential for clinical translation, we selected deferasirox (DFS), an FDA‐approved drug, to specifically target the hyphae morphology of *C. albicans*. In this study, we combined RAP and DFS, leveraging their respective strengths, to repurpose existing medications and employ synthetic lethal therapy tailored to the dimorphism of *C. albicans*, aiming to achieve superior therapeutic outcomes. We found that DFS can limit the iron resource to destroy the vacuole structure of *C. albicans* and cause the metabolism of ribosome‐related amino acids to be disordered, thus inhibiting the function of ribosomes and reducing the number of ribosomes. Additionally, DFS inhibits the polarization process involved in hyphae elongation, effectively limiting hyphae growth through these two mechanisms. On the other hand, RAP eliminates yeast‐form *C. albicans* by inhibiting the mTOR pathway. Notably, the stimulatory effect of RAP on hyphae development, mediated through the activation of *HDA1*, is mitigated by DFS (**Figure** **1**). Finally, we successfully integrated RAP and DFS into a single pharmaceutical preparation utilizing bionics technology. It enhanced the targeting of the drugs toward *C. albicans* while mitigating the adverse effects of RAP on immune cells. Consequently, this integrated nano‐preparation significantly improved the survival rate of mice with systemic *C. albicans* infections. Notably, this synthetic lethal therapy based on dimorphism also demonstrated an ideal therapeutic effect against FLU‐resistant type *C. albicans* infection.

## 2 Results

### 2.1 The Therapeutic Effect on Yeast of *C. albicans*

**Figure 2:**
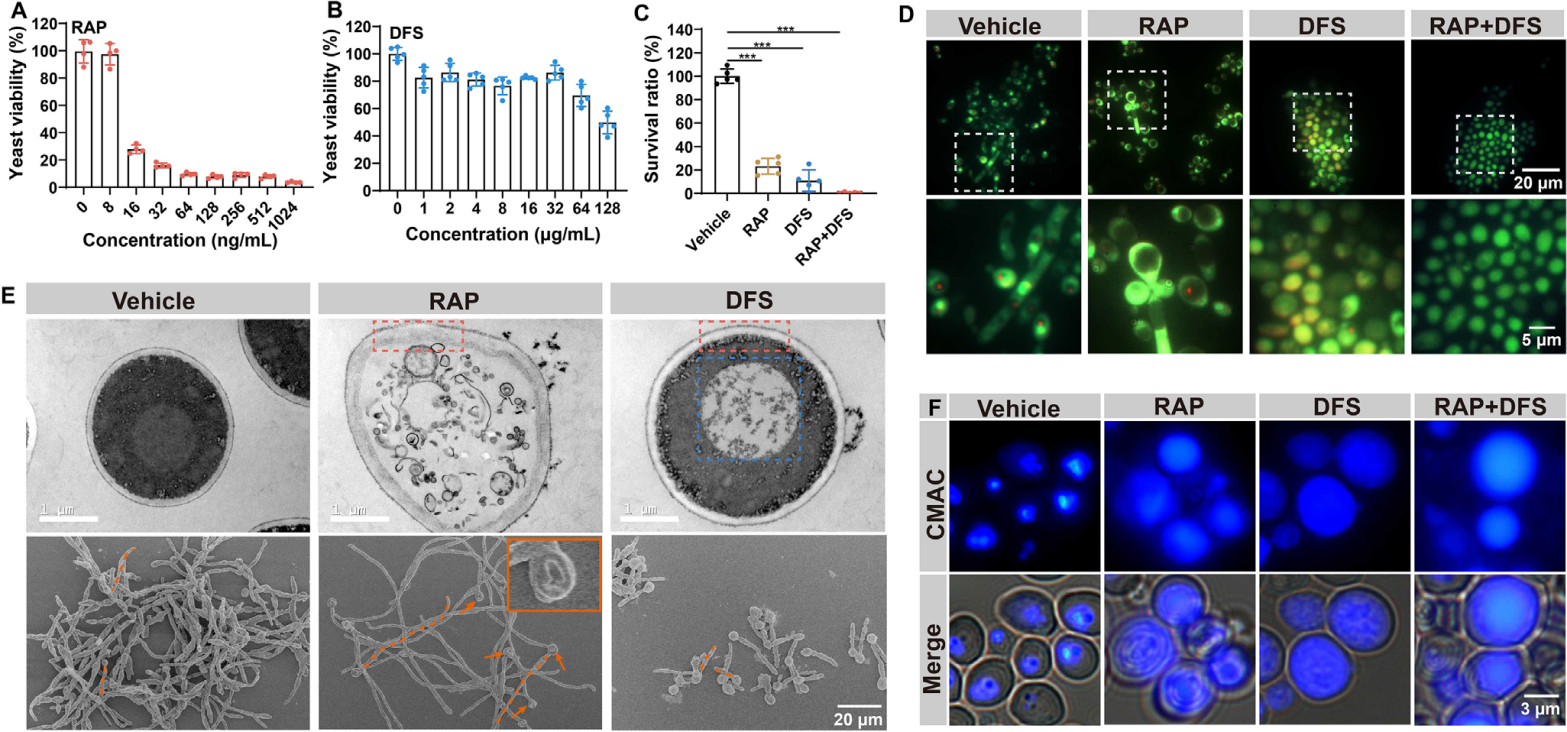
The therapeutic effects of RAP and DFS on yeast cells. A,B) The yeast viability after RAP (A) and DFS (B) treatment. (*n* = 4,5). C,D) The quantification of cell survival ratio and the images of live *C. albicans* stained by FUN 1 after various treatments (scale bars = 20 µm and 5 µm, *n* = 5). E) The images of transmission electron microscope and scanning electron microscope of *C. albicans* after different treatments. The red dashed box represents the cell membrane structure, and the blue dashed box represents the vacuole structure. The orange dashed line indicates the intact hyphae of single *C. albicans*, and the orange arrow points to the collapsed yeast. (scale bars = 1 µm and 20 µm) F) The representative images of the vacuole cavity stained by Cell Tracker Blue CMAC (scale bars = 3 µm). Data are shown as mean ± SD; ***p < 0.001.

The target of RAP in combating *C. albicans* diverges from most antifungal medications. We evaluated the growth inhibition effect of RAP on *C. albicans* using a minimal inhibitory concentration (MIC) experiment. The results showed that RAP could suppress the growth of yeast *C. albicans* at a concentration of 16 ng mL^−1^, and the survival rate of *C. albicans* was less than 10% at a concentration of 64 ng mL^−1^ (**Figure** **2**A). Compared with the existing anti‐*C. albicans* drugs, including FLU (4000 ng mL^−1^) and amphotericin B (250 ng mL^−1^), RAP shows a better therapeutic effect (Figure S1A,B, Supporting Information). Furthermore, DFS targeting hyphae form exhibited a noticeable anti‐*C. albicans* effect only when administered at high concentrations. At lower concentrations, its inhibitory effect diminished to less than 20% (Figure 2B). FUN‐1 staining is used to assess the viability of yeast cells. In live cells, the FUN‐1 probe accumulates in vacuoles and forms distinct red fluorescent puncta. Following treatment with RAP, the number of red fluorescent puncta was markedly reduced, indicating that RAP exhibits satisfied fungicidal activity against yeast cells. Similarly, DFS treatment also resulted in a significant reduction in red fluorescence puncta, suggesting that high concentrations of DFS possess potent yeast‐killing effects. When these two drugs were combined, almost all red fluorescence puncta diminished, indicating that RAP and DFS may kill yeast through distinct mechanisms (Figure 2C,D). Subsequently, we employed transmission and scanning electron microscopes to delve deeper into the microscopic alterations in *C. albicans* post‐drug administration. Upon treatment with RAP, the integrity of the vacuole structure and cell membrane of *C. albicans* is compromised, leading to leakage of cellular contents. Consequently, the *C. albicans* cells in the RAP group turn to an ellipsoidal shape, mirroring the localized collapse visible under the scanning electron microscope. In stark contrast to the RAP group, the cytoplasm contents of *C. albicans* in the DFS group did not exhibit significant leakage; instead, only alterations in vacuole contents were observed. Furthermore, scanning electron microscopy indicated that RAP spurred the hyphae formation in *C. albicans*, potentially linked to the organism's self‐defense mechanism. Conversely, DFS demonstrated a notable inhibitory effect on hyphae formation (Figure 2E). In the DFS‐treated group, FUN‐1 staining in yeast cells partially appeared yellow. Electron microscopy results suggest that this yellow coloration is due to DFS‐induced disruption of vacuolar structures, while the cell membrane remains intact. This disruption may alter the cytoplasmic microenvironment, causing the FUN‐1 probe to display diffuse red fluorescence throughout the cytoplasm. The overlap of this diffuse red fluorescence with green fluorescence produces a yellow appearance (Figure 2D). The specific damage inflicted by DFS on vacuoles is likely to hinder the growth of hyphae.^[^ ^15^, ^16^ ^]^ Therefore, we conducted a more thorough investigation into the structural integrity of vacuoles. In the vehicle group, the vacuole cavity dye CMAC aggregated within the vacuole structure. However, following treatment with RAP or DFS, the dye exhibited a diffuse distribution, further confirming that the vacuole's integrity had been disrupted (Figure 2F).

### 2.2 Inhibition of Hyphal Growth and Invasion of *C. albicans* by DFS

**Figure 3:**
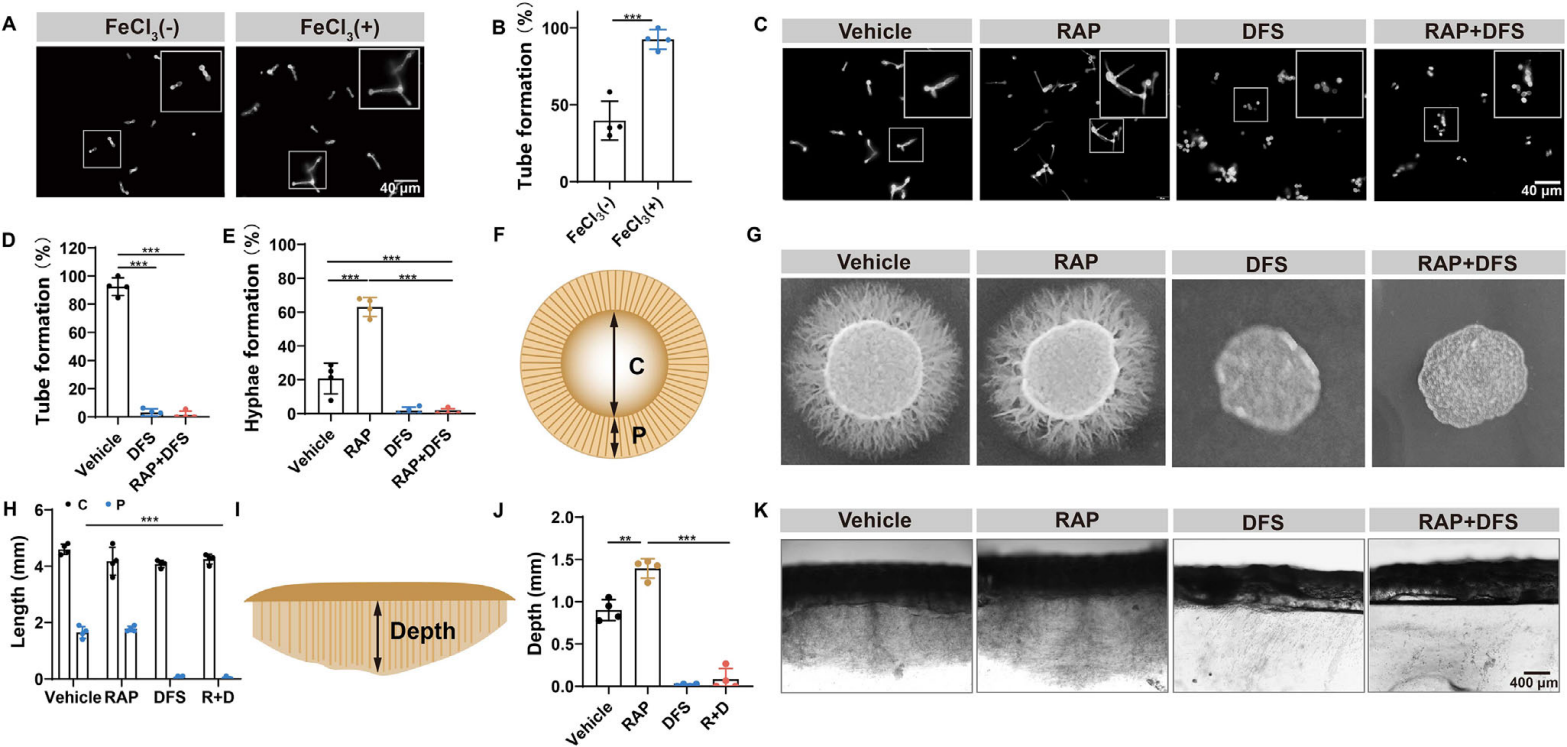
Inhibitory effect of DFS on hyphal form. A,B) The morphology of *C. albicans* strains stained with calcofluor white in liquid medium supplemented with FeCl_3_ (scale bars = 40 µm) and quantification of tube formation rate from (A) (*n* = 4). C) The morphology of *C. albicans* strains in liquid medium after various treatments (scale bar = 40 µm). D,E) Quantification of tube formation and hyphae formation rate from (C) (*n* = 4). F) The illustration of a top view of the hyphal colony on solid agar. The diameter of the center part without hyphae was defined as C. The distance of visible hyphal part scattering around the central region was defined as P. G) The hyphal morphology on solid media containing different medicines. H) Quantification of the C and P values from (G) (*n* = 4). I) The schematic diagram of the vertical section of the hyphal colony on solid agar. Depth is the maximum distance of hyphae penetrating agar medium from the surface. J,K) Quantification of the depth from (K) and vertical sections images of hyphal colonies on solid media obtained by microscopy (scale bars = 400 µm, *n* = 4). Data are shown as mean ± SD; **p < 0.01; ***p < 0.001.

During an unplanned experiment, we discovered a potential link between the formation of hyphae in *C. albicans* and the availability of iron ions. Iron ions are essential nutrients for the growth of *C. albicans*. To our knowledge, no prior studies have explored the relationship between hyphae formation and iron supply. To investigate this further, we added FeCl_3_ to the culture medium. The results revealed that FeCl_3_ had a stimulatory effect on the hyphal formation of *C. albicans* (**Figure** **3A,B**; Figure S2A,B, Supporting Information). Correspondingly, the biofilm formation of *C. albicans* increased significantly (Figure S2C, Supporting Information). After treating *C. albicans* with DFS, the intracellular iron ion content decreased significantly. This observation suggests that DFS reduces iron ion uptake by *C. albicans*, likely by limiting access to iron sources (Figure S3A,B, Supporting Information). When *C. albicans* was in the hyphae‐inducing conditions, namely RPMI 1640 culture medium at 37 °C, it transformed yeast morphology to hyphae morphology through budding. However, after DFS treatment, ≈97% of *C. albicans* remained in the yeast form. Notably, in combination with RAP and DFS, the inhibition rate rose to 95% (Figure 3C,E). To simulate the morphological transformation process of *C. albicans* in vivo, we cultured *C. albicans* in RPMI 1640 solid medium containing different drugs. We measured the diameter of the central region in the colony (C) and determined the distance between hyphal insertion and the edge of the central region (P) (Figure 3F). After ten days of cultivation, the vehicle group exhibited the growth of numerous radial hyphae extending beyond the central area, successfully achieving the invasion of “distal tissue”. Following DFS treatment, the peripheral region of the colony underwent a significant reduction, with virtually no observable hyphae structures present (Figure 3G,H). To further assess the drug's efficacy in controlling deep tissue invasion, we conducted additional observations on the infiltration of hyphae within an agar medium. The results indicated that RAP significantly promoted the deep invasion of *C. albicans*. The DFS group and the DFS+RAP group exhibited virtually no hyphae infiltration into the agar. Notably, DFS not only effectively suppressed the formation of *C. albicans* hyphae morphology but also counteracted the enhancement of invasion induced by RAP (Figure 3I–K).

### 2.3 The Transcriptomics Discrepancy of *C. albicans* after RAP and DFS Treatment

**Figure 4:**
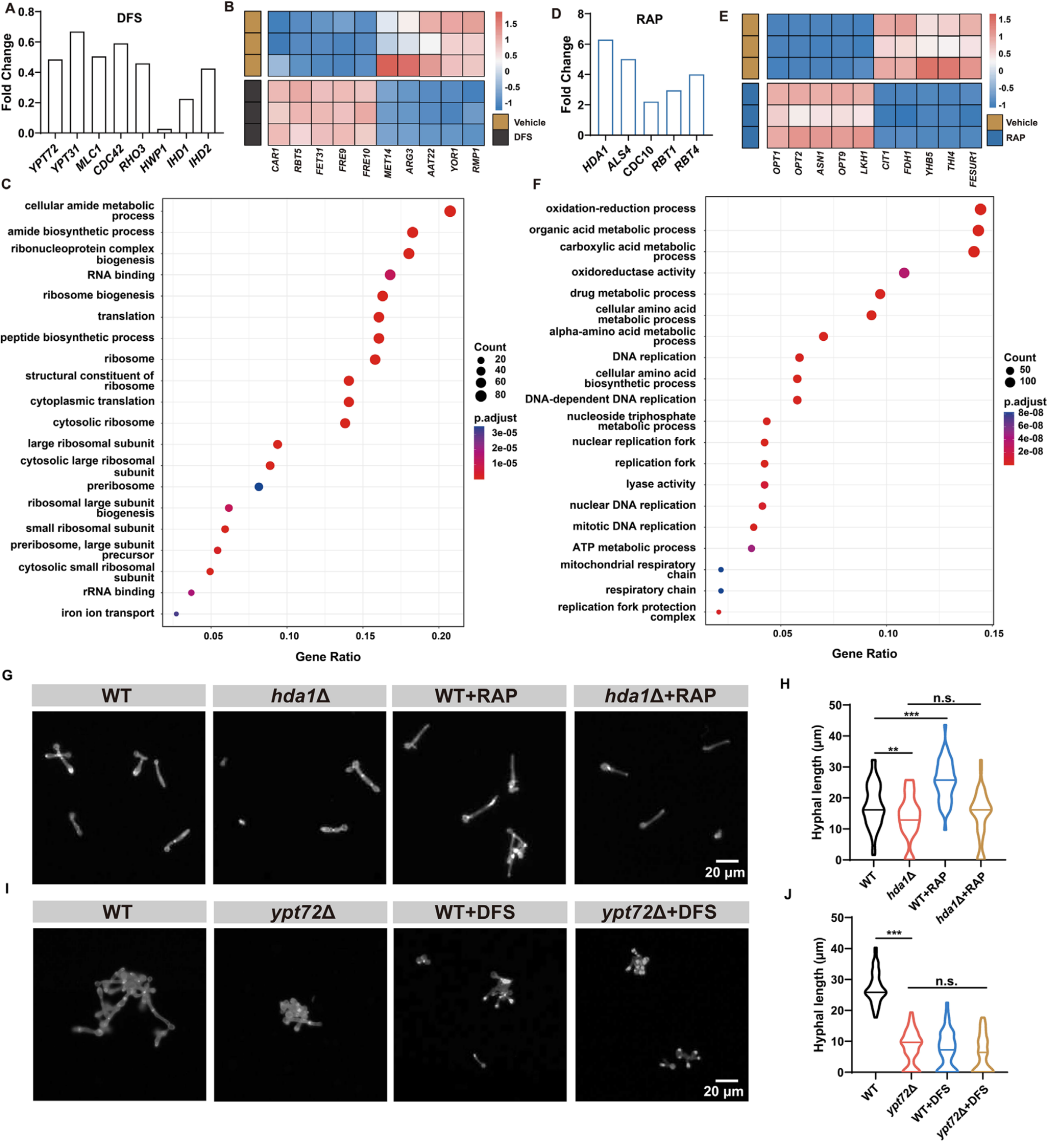
The transcriptomics analysis of *C. albicans* after RAP and DFS treatment. A) The altered genes associated with hyphal formation after DFS treatment. B) The heat map of changed genes after DFS treatment. The red squares indicate the increased transcription of related genes (*n* = 3). C) GO pathway analysis. The size and color of the filled circle represent the *p*‐value and the number of differential genes enriched in this pathway, respectively. D) Representative genes associated with hyphal formation after RAP treatment. E) The heat map of changed genes after RAP treatment (*n* = 3). F) GO pathway analysis. G) The morphology of different *C. albicans* strains (WT and *hda1*Δ) in liquid medium after various treatments (scale bar = 20 µm). H) Quantification of hyphal length from (G), the horizontal line represents the median value (*n* = 50). I) The morphology of different *C. albicans* strains (WT and *ypt72*Δ) in liquid medium after various treatments (scale bar = 20 µm). J) Quantification of hyphal length from (I), the horizontal line represents the median value (*n* = 50). Data are shown as mean ± SD; **p < 0.01; ***p < 0.001. n.s. indicates non‐significance.

RAP can inhibit the growth and metabolism of yeast after inhibiting mTOR, but why can it promote hyphae growth? DFS inhibits hyphae growth through iron chelation. How exactly does it work? To further investigate the therapeutic disparities between RAP and DFS in treating *C. albicans* infection, we utilized transcriptomics to analyze the transcriptional alterations in *C. albicans* following exposure to DFS or RAP (Figure S4A,B, Supporting Information). After DFS chelates iron, the transcription of membrane fusion regulator *YPT72* was decreased, which may lead to vacuole damage.^[^ ^17^ ^]^ The levels of *YPT31, MLC1, CDC42*, and *RHO3* related to vesicle extreme transport during hyphal polarization were significantly down‐regulated.^[^ ^18^ ^]^ The down‐regulation of hyphae‐specific genes *HWP1, IHD1*, and *IHD2* further proved the hyphae inhibition of DFS (**Figure** **4**A).^[^ ^19^ ^]^

The thermogram showed that after DFS treatment, the transcription level of iron uptake‐related genes (*FET31, FRE10, FRE9, RBT5*) increased, and this change was often related to the iron‐restricted environment, which indicated that iron deficiency and iron ion disorder might occur in *C. albicans* cells.^[^ ^20^, ^21^, ^22^ ^]^ The destruction of the vacuole structure led to an amino acid metabolism disorder. *ARG3* encoding ornithine carbamoyltransferase was down‐regulated, which may inhibit arginine biosynthesis. The up‐regulation of *CAR1* encoding arginase promotes the hydrolysis of arginine. The decrease of *MET14* may inhibit the biosynthesis of cysteine and methionine. The down‐regulation of *AAT22* will also inhibit the biosynthesis of arginine, tyrosine, tryptophan, and other amino acids. The down‐regulation of *RMP1* can inhibit the ribonuclease MRP protein subunit RMP1, thus inhibiting the function and quantity of ribosomes. In addition, the transcription level of *YOR1* encoding an ABC transporter decreased, which may increase the sensitivity of *C. albicans* to other ABC transporter substrate drugs (Figure 4B).^[^ ^23^ ^]^ GO analysis showed that the iron ion transport of *C. albicans* was affected by DFS. The structural composition and physiological function of ribosomes, such as ribonucleoprotein complex biogenesis, the structural constitution of the ribosome, RNA binding, ribosome biogenesis, and translation, have changed, which may be due to the inhibition of the synthesis of ribosomal proteins such as arginine, thus inhibiting the number and function of ribosomes. Ribosomal proteins have a high cell abundance, accounting for ≈6% of the protein group, among which arginine content is high.^[^ ^24^ ^]^ The transcription levels of numerous genes encoding the ribosomal 40S and 60S subunits were also markedly downregulated (Tables S1,S2, Supporting Information). The inhibition of protein translation in ribosomes will further limit the extension of hyphae and the division of yeast morphology (Figure 4C).^[^ ^25^, ^26^ ^]^ Similarly, KEGG analysis showed that arginine biosynthesis, coenzyme synthesis, amino acid metabolism, lipid metabolism, and nucleic acid metabolism‐related pathways have changed. The genes related to meiosis in yeast have also changed, which is why DFS has a certain inhibitory effect on yeast morphology (Figure S4C, Supporting Information).

Following RAP treatment, the transcription of the deacetylase gene *HDA1*, intimately associated with hyphae formation, increased substantially.^[^ ^27^ ^]^ Furthermore, the transcription levels of hyphae‐specific genes (*ALS4, CDC10, RBT1, RBT4*) were significantly up‐regulated (Figure 4D).^[^ ^19^ ^]^ After RAP treatment, the transcription of *ASN1* encoding asparagine synthase (glutamine‐hydrolysis) was increased, which might promote the hydrolysis of glutamine. Involved in amino acid synthesis, the transcription levels of *THI4* encoding vitamin B1 and *YHB5* encoding vitamin B6 were down‐regulated, which regulated cell catabolism. The transcription levels of *CIT1* encoding tricarboxylic acid cycle rate‐limiting enzyme citrate synthase and *FDH1* gene controlling coenzyme regeneration in the redox system were significantly down‐regulated, which indicated that RAP restricted the anabolism and catabolism of *C. albicans* at the same time, and could inhibit the division and proliferation of *C. albicans*. In negative feedback, *OPT1, OPT2*, and *OPT9*, responsible for amino acid intake and nutrient acquisition, were up‐regulated, which may also be a crucial reason why RAP promoted hyphae formation (Figure 4E).^[^ ^28^ ^]^ In GO analysis, after RAP treatment, organic acid metabolic processes, carboxylic acid metabolic processes, amino acid synthesis, and metabolism have changed significantly, closely related to the regulation of glutamine and other amino acids by the mTOR pathway.^[^ ^29^ ^]^ The disturbance of the oxidation–reduction process, ATP metabolic process, nucleic acid metabolism, DNA replication, and other processes involved in the metabolic process are why RAP kills yeast morphology. The changes in genes related to drug metabolic processes also indicate that RAP can change the sensitivity of *C. albicans* to drugs (Figure 4F). KEGG analysis also showed that the pathways related to oxidative phosphorylation, biosynthesis and metabolism of amino acids, carbon metabolism, and DNA replication changed significantly after RAP treatment, which further verified the killing effect of RAP on yeast morphology (Figure S4D, Supporting Information).

To further investigate the effects of RAP and DFS on the *HDA1*, *YPT72*, and *TOR1* genes in *C. albicans*, we separately knocked out the *HDA1*, *YPT72*, and *TOR1* genes in this fungus using homologous recombination. The knockout of *HDA1*, *YPT72*, and *TOR1* was verified by colony PCR (Figure S5, Supporting Information). The hyphal length of the *hda1*Δ mutant was significantly shorter than that of the wild‐type (WT) *C. albicans*, because *HDA1* plays a key role in the elongation stage of hyphal growth. Knockout of *HDA1* thus restricted hyphal elongation. Upon treatment with RAP, the hyphae of the WT *C. albicans* exhibited significant elongation, whereas the hyphal length of the *hda1*Δ mutant remained comparable to that of the mutant without RAP treatment. This indicates that RAP is unable to promote hyphal elongation when it loses *HDA1* as a target (Figure 4G,H). Given that RAP predominantly targets the mTOR pathway, we delved deeper to investigate whether the hyphal‐promoting action of RAP was associated with this particular pathway. Employing homologous recombination, we successfully knocked out the *TOR1* genes in this fungal species. It was observed that the hyphal length of the *tor1*Δ mutant was notably shorter compared to that of the WT *C. albicans*. Notably, RAP treatment induced hyphal elongation in both strains, leading to comparable hyphal lengths between the two groups. These findings clearly demonstrate that RAP does not facilitate hyphal growth via the inhibition of the mTOR pathway (Figure S6A,B, Supporting Information). Under hypha‐inducing conditions, the hyphal length of the *ypt72*Δ mutant was significantly shorter than that of the WT *C. albicans*. Knockout of *YPT72* may impair vacuolar structure, thereby restricting hyphal development. Following DFS treatment, the hyphal length of WT *C. albicans* was significantly reduced, whereas that of the *ypt72*Δ mutant remained unchanged and was comparable to the WT *C. albicans* treated by DFS. These findings indicate that DFS cannot exert its hyphal‐inhibiting effect once *YPT72* is knocked out (Figure 4I,J). Interestingly, DFS did not hinder hyphae growth by disrupting the conventional hyphae growth pathway (Table S3, Supporting Information). It limited the source of iron in cells. On one hand, DFS restricted the polarization process of hyphae growth. On the other hand, it impaired vacuoles by inhibiting *YPT72*, thereby disrupting the metabolism of amino acids essential for ribosomal proteins. This led to a suppression of ribosomal function and quantity, ultimately limiting the provision of proteins necessary for hyphae growth. RAP inhibited the metabolism and proliferation of yeast through the suppression of the mTOR pathway. The upregulation of *HDA1* is associated with RAP's role in promoting hyphae formation.

### 2.4 Fabrication and characterization of nano‐preparation for synthetic Lethal therapy

**Figure 5:**
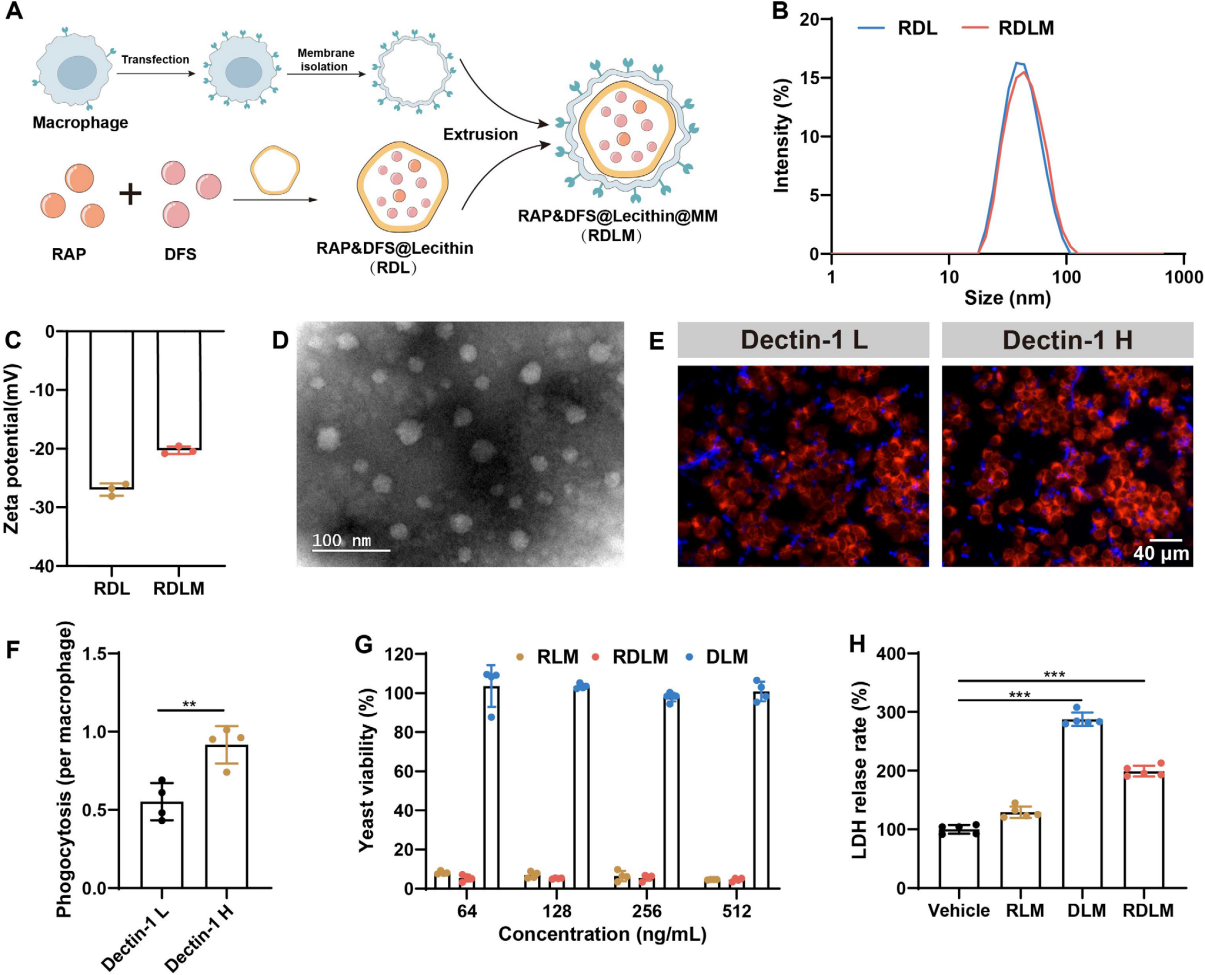
Preparation and characterization of nanomedicine for synthetic lethal therapy. A) The illustration of the preparation process of RDLM. B,C) Size distribution and zeta potential of RDL and RDLM (*n* = 3). D) Transmission electron microscope image of RDLM (scale bar = 100 nm). E) Representative images of two types of macrophages incubated with *C. albicans* for 2 h (scale bar = 40 µm, *n* = 4). L, Low expression of dectin‐1; H, High expression of dectin‐1. F) Phagocytosis of *C. albicans* per a macrophage. G) The viability of yeast form after different treatments of various concentrations (*n *= 4). H) Lactate dehydrogenase released from the hyphal form after different treatments (*n* = 5). Data are shown as mean ± SD; **p < 0.01; ***p < 0.001.

To address the challenges posed by drug resistance in existing antifungal medications and systemic infections caused by dimorphism of *C. albicans*, we combined RAP and DFS to achieve biphasic synthetic lethal therapy. Furthermore, we utilized nanotechnology to formulate these two drugs into an integrated pharmaceutical preparation, enhancing drug targeting and thus improving drug utilization efficiency while minimizing side effects.^[^ ^30^ ^]^ RAP and DFS were coated with lecithin, which has good biocompatibility, to form nanovesicles. Rapamycin @ lecithin nanoparticles (RL), Deferasirox @ lecithin nanoparticles (DL), Rapamycin & Deferasirox @ lecithin nanoparticles (RDL), and Rapamycin and Deferasirox @ lecithin nanoparticles @ macrophage membrane (RDLM) were obtained in the same manner. To enhance the targeted delivery of nano‐vesicles, we employed engineered macrophage membranes with high expression of dectin‐1 to encapsulate the nano‐vesicles RDL, resulting in RDLM. This approach may improve the targeting efficiency of the drug preparations toward *C. albicans* while mitigating the adverse effects of RAP on immune cells (**Figure** **5**A). The dectin‐1 receptor on the macrophage membrane can specifically target β‐glucan on the *C. albicans* cell wall.^[^ ^31^, ^32^ ^]^ By adjusting the concentration of lecithin, ultrasonic emulsification time, and power, we finally got RDL with a particle size of ≈40 nm. After coating the macrophage membrane, the particle size had no significant change (Figure 5B; Figure S7A, Supporting Information). The zeta potential of RDL changed from −26.8 to −19.5 mV after being coated with macrophage membrane, which preliminarily proved the coating of macrophage membrane (Figure 5C). The zeta potentials of RDL, DL, and RL were similar (Figure S7B, Supporting Information). RDL and RDLM were nearly spherical and evenly distributed, and their sizes were similar to DLS results (Figure 5D; Figure S7C, Supporting Information). Since the dectin‐1 expression on the surface of resting macrophages is limited, we further augmented the dectin‐1 level through lentiviral transfection. Here, the puro resistance gene was utilized for cell screening, while EGFP served as a marker to detect protein expression. After screening multiple rounds, the purity of the engineered macrophages exceeded 90% (Figure S8A,B, Supporting Information). Macrophages with high expression of dectin‐1 had stronger phagocytosis of *C. albicans* than wild‐type macrophages (Figure 5E,F). Furthermore, when macrophages with high dectin‐1 expression were incubated with *C. albicans*, they significantly decreased the hyphae formation. These macrophages are also more prone to phagocytose yeast forms of *C. albicans*, suggesting that dectin‐1 plays a crucial role in the elimination of *C. albicans* by macrophages (Figure S8C,D, Supporting Information). Both RLM and RDLM exhibit a notable inhibitory effect on the growth of *C. albicans* yeast. At a RAP concentration of 64 ng mL^−1^, the inhibitory activity of both RLM and RDLM toward *C. albicans* remains above 90%, comparable to that of free RAP. However, the concentration of DFS in DLM is below the MIC for yeast‐form *C. albicans*, resulting in no inhibitory effect on yeast‐form *C. albicans* in the DLM group (Figure 5G). Compared with the RLM group, the DLM and RDLM groups significantly increased lactate dehydrogenase released by hyphal *C. albicans*, indicating that they had a better hyphal state destruction effect (Figure 5H). Therefore, the integrated nano‐preparation RDLM can significantly kill and destroy yeast‐type and hyphae‐type *C. albicans*.

### 2.5 Mitigating the Damage of RAP to Macrophage

**Figure 6:**
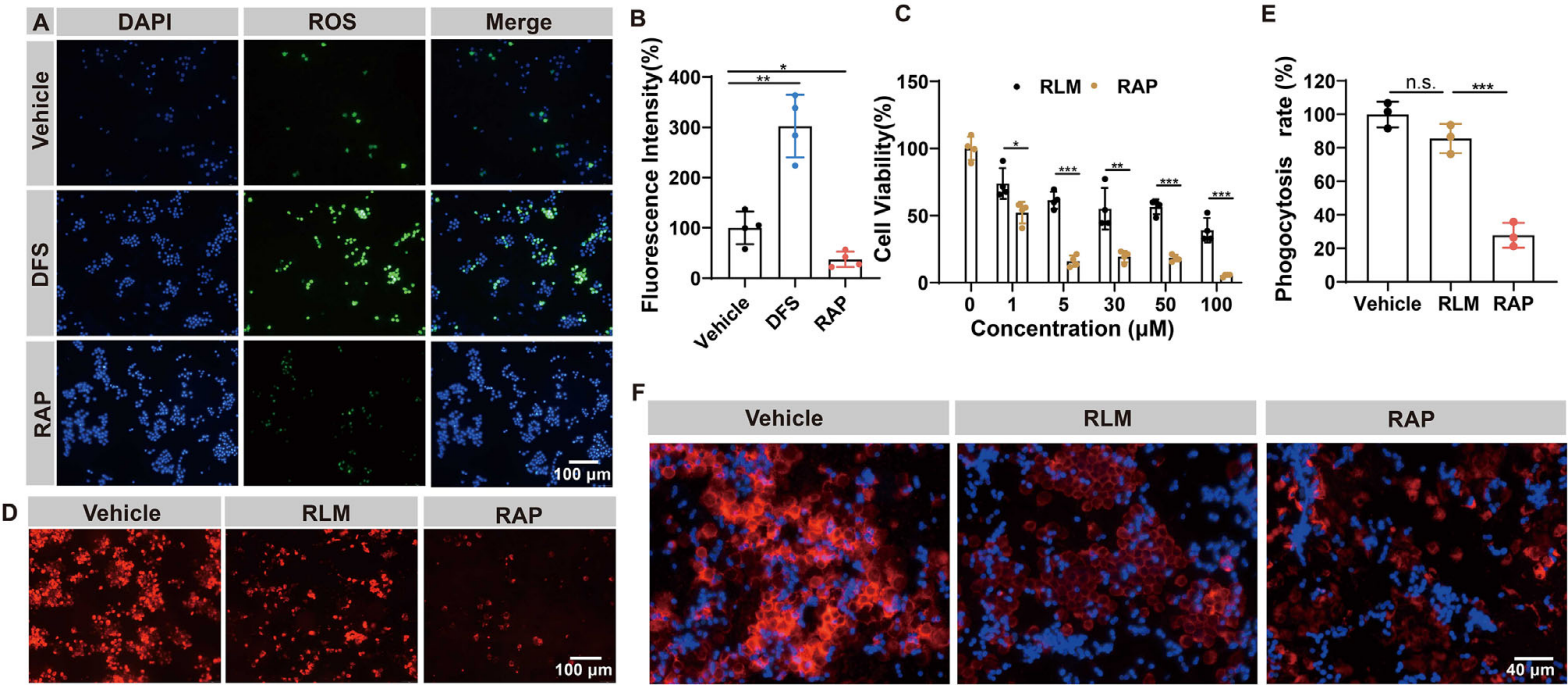
Mitigating the damage of RAP to macrophage by RLM. A) Imaging of ROS in macrophage stained by DCFH‐DA after different treatments (green fluorescence). The nucleus is indicated by DAPI ([RAP] = 16 ng mL^−1^, [DFS] = 256 µg mL^−1^; scale bar = 100 µm). B) The quantification of DCFH‐DA fluorescence intensity (*n *= 4). C) The cytotoxicity of RAP and RLM to RAW264.7 cells (*n* = 4). D) Imaging of macrophages after different treatments. The cell membrane is indicated by DID (red; scale bar = 100 µm; [RAP] = 10 µm). E,F) Quantification of phagocytosis rate and images of macrophages incubated with *C. albicans* for 2 h. *C. albicans* was indicated by calcofluor white (blue) and macrophages were stained with DID (red). Macrophages were priorly exposed to RAP or RLM for 24 h ([RAP] = 10 µm; scale bar = 40 µm, *n* = 3). Data are shown as mean ± SD; *p < 0.05; **p < 0.01; ***p < 0.001. n.s. indicates non‐significance.

The failure of RAP in fungal treatment is due to immunosuppressive properties, particularly its adverse impact on macrophage function.^[^ ^33^ ^]^ Macrophages can phagocytose yeast forms of *C. albicans* and eliminate them through reactive oxygen species (ROS) production.^[^ ^34^, ^35^, ^36^ ^]^ *C. albicans* was pre‐treated with various drugs and then incubated with macrophages. The macrophages in the DFS group produced a significant amount of ROS. This suggests that DFS maintained *C. albicans* in its yeast form by inhibiting hyphae growth, making it more susceptible to phagocytosis by macrophages. RAP promoted hyphae growth, leading to macrophages producing only a small amount of ROS, just 37.2% of the vehicle group's level. DFS may improve the phagocytosis and the killing efficacy of macrophages against *C. albicans* in vivo (**Figure** **6**A,B). Furthermore, free RAP can disrupt the morphology of macrophages and inhibit their growth. At a concentration of 5 µg mL^−1^, the survival rate of macrophages dropped to only 15.9%. However, RLM, modified through bionic nanotechnology, can significantly mitigate the toxic effects of RAP on macrophages (Figure 6C,D). To investigate the impact of RAP on macrophage function, macrophages were pre‐treated with RAP or RLM and then incubated with *C. albicans*.^[^ ^37^ ^]^ The phagocytosis of macrophages in the RAP group was reduced to 27.9%. There was no significant difference in the phagocytosis of *C. albicans* by macrophages treated with RLM compared to the vehicle group (Figure 6E,F). Therefore, applying bionic nanotechnology can significantly mitigate the damage caused by RAP to macrophages, while DFS can also enhance the ability of macrophages to eliminate exogenous pathogens.

### 2.6 The Improved Therapeutic Effect of Synthetic Lethal Therapy for Systemic Infection

**Figure 7:**
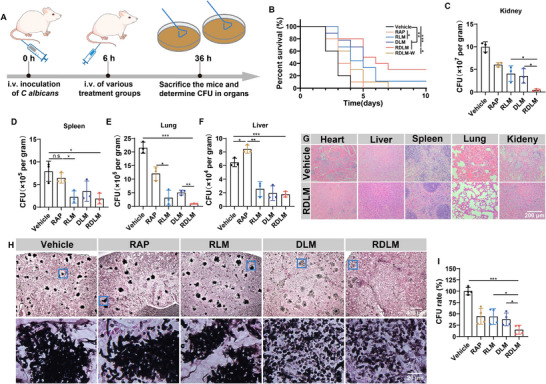
The therapeutic effect of synthetic lethal therapy for systemic infection. A) Experimental procedures for assessing antifungal efficacy in the model of *C. albicans* systemic infection. B) Survival curve of infected mice after various treatments (*n* = 9,10). C–F) The CFU of *C. albicans* in the kidney (C), spleen (D), lung (E), and liver (F). The tissues were harvested from the infected tissues of mice at 30 h after the different treatments (*n* = 3). G) Representative H&E staining images of tissue sections from infected mice treated with PBS or RDLM (scale bar = 200 µm). H) Representative periodic acid‐silver methenamine (PASM) staining images of kidney tissue from infected mice after different treatments (scale bar = 400 µm). The images in the second line are an enlargement of the blue box in the first line (scale bar = 20 µm). I) Relative quantification of flora in the kidney (*n* = 4). Data are shown as mean ± SD; *p < 0.05; **p < 0.01; ***p < 0.001. n.s. represented non‐significance.

Next, a systemic infection model was established through intravenous injection of *C. albicans* and then randomly divided into six groups: the vehicle group, RAP group, RLM group, DLM group, RDLM‐W group (RDL coated with wild‐type macrophage membrane), and RDLM group. The model mice were administered different medications via tail vein injection, and their survival time was documented. In a separate experiment, model mice were euthanized 36 h post‐modeling to assess the distribution of *C. albicans* in various organs following drug treatment (**Figure** **7**A). All model mice in the vehicle group succumbed within 4 days, while there was no notable difference in survival time between the RAP group and the vehicle group, suggesting that free RAP alone could not exert an antifungal effect against *C. albicans*. However, the survival rate of mice in the RLM group was 44.4%, indicating that RAP may exert some antifungal effect by mitigating damage to macrophages. On the 4th day, the survival rate of mice in the RDLM group was 80%, and even on the 10th day, the survival rate in the RDLM treatment group remained at 30%. Notably, the surviving mice in the RDLM group maintained good health until the end of the feeding period (20 days). Conversely, the therapeutic effect of RDLM‐W on infected mice was not satisfactory, suggesting that macrophage membranes with high dectin‐1 expression play a superior synergistic and mitigating role when targeting the β‐glucan of *C. albicans* (Figure 7B). We performed periodic acid‐silver methenamine (PASM) and periodic acid‐schiff (PAS) pathological staining analyses on mice in the RDLM group that survived for 14 days. No fungal colonies or hyphae were observed in the kidneys, the primary target organ of *C. albicans* infection. This finding indicates that *C. albicans* in the kidneys was completely eradicated, demonstrating that RDLM exerts a certain long‐term therapeutic effect (Figure S9A, Supporting Information).

After 30 h of treatment with different pharmaceutical preparations, we measured the *C. albicans* colony load in the liver, spleen, lung, and kidney of mice with systemic infections using candida chromogenic solid medium. Compared to other treatment groups, RDLM significantly reduced the fungal load in these organs, clearing over 95% of *C. albicans* in the kidneys and lungs. Across the four organs, the therapeutic effects of the five groups showed some similarity, with RDLM performing best, followed by RLM and DLM. RAP exhibited some therapeutic effect, but notably, the *C. albicans* load in the liver of the RAP group was higher than that in the vehicle group, and the load in the spleen of both groups was comparable. This is closely associated with the damage of RAP to these two organs, which are rich in macrophages (Figure 7C–F; Figure S9B, Supporting Information). H&E staining showed that the direct damage to the heart, spleen, kidney, and lung tissues by *C. albicans* was significantly reduced after RDLM treatment (Figure 7G). To observe the morphology of *C. albicans* within organs, we stained kidney sections using hexamine silver staining. The number of fungal colonies in the RDLM group was only 14.9% of that in the vehicle group, which aligns with the statistical results obtained from plating. More importantly, RDLM effectively inhibited the hyphal growth and invasion of *C. albicans* in tissues, maintaining the remaining *C. albicans* in the yeast state. In contrast, similar to the experimental results obtained in vitro, treatment with RAP and RLM promoted the growth of *C. albicans* hyphae in the organs (Figure 7H,I). In addition, in the severe acute infection model of mice, we found that RDLM can still significantly improve the survival time of mice (Figure S9C,D, Supporting Information). Therefore, RDLM achieved synthetic lethal treatment by targeting *C. albicans* and concurrently interfering with its two‐phase morphology, thus significantly prolonging the survival time of mice with systemic infections.

To evaluate the clinical application potential of RDLM, we investigated the biodistribution and potential toxic side effects of RDLM. In vivo imaging results showed that the concentration of RDLM‐IR775 in major organs such as the liver and kidney reached a peak at ≈12 h, with a significantly higher level than that of free IR775. Ex vivo organ tissue analysis revealed that compared with the free drug, the concentration of RDLM in the kidneys was significantly increased. This phenomenon may be attributed to the fact that the kidneys of mice in the infection model exhibit the highest fungal burden; RDLM can bind to *C. albicans* more easily via dectin‐1, thereby accumulating in the kidneys (Figure S10A–C, Supporting Information). We determined the plasma drug concentrations of free DFS and RDLM at different time points. The half‐life of RDLM was 5 h, which was 16.7 times that of free DFS. The area under the curve of RDLM was 10.45 µg h mL^−1^, 12.2 times that of free DFS. These results indicate that RDLM can significantly improve the pharmacokinetic profile of the free drug and prolong its half‐life in the bloodstream (Figure S10D, Supporting Information). Compared with healthy mice, there were no significant changes in the levels of blood urea nitrogen (BUN), creatinine (CRE), alanine aminotransferase (ALT), aspartate transaminase (AST), and total bilirubin (TB) in the serum of mice injected with RDLM. This result indicates that the functions of the liver and kidneys were not significantly affected (Figure S10E–I, Supporting Information). H&E pathological analysis revealed no observable damage in the heart, liver, spleen, lung, or kidney. Therefore, RDLM exhibits a favorable safety profile and certain potential for clinical application (Figure S10J, Supporting Information).

### 2.7 Synthetic Lethal Therapy for Fluconazole‐Resistant *C. albicans*

**Figure 8:**
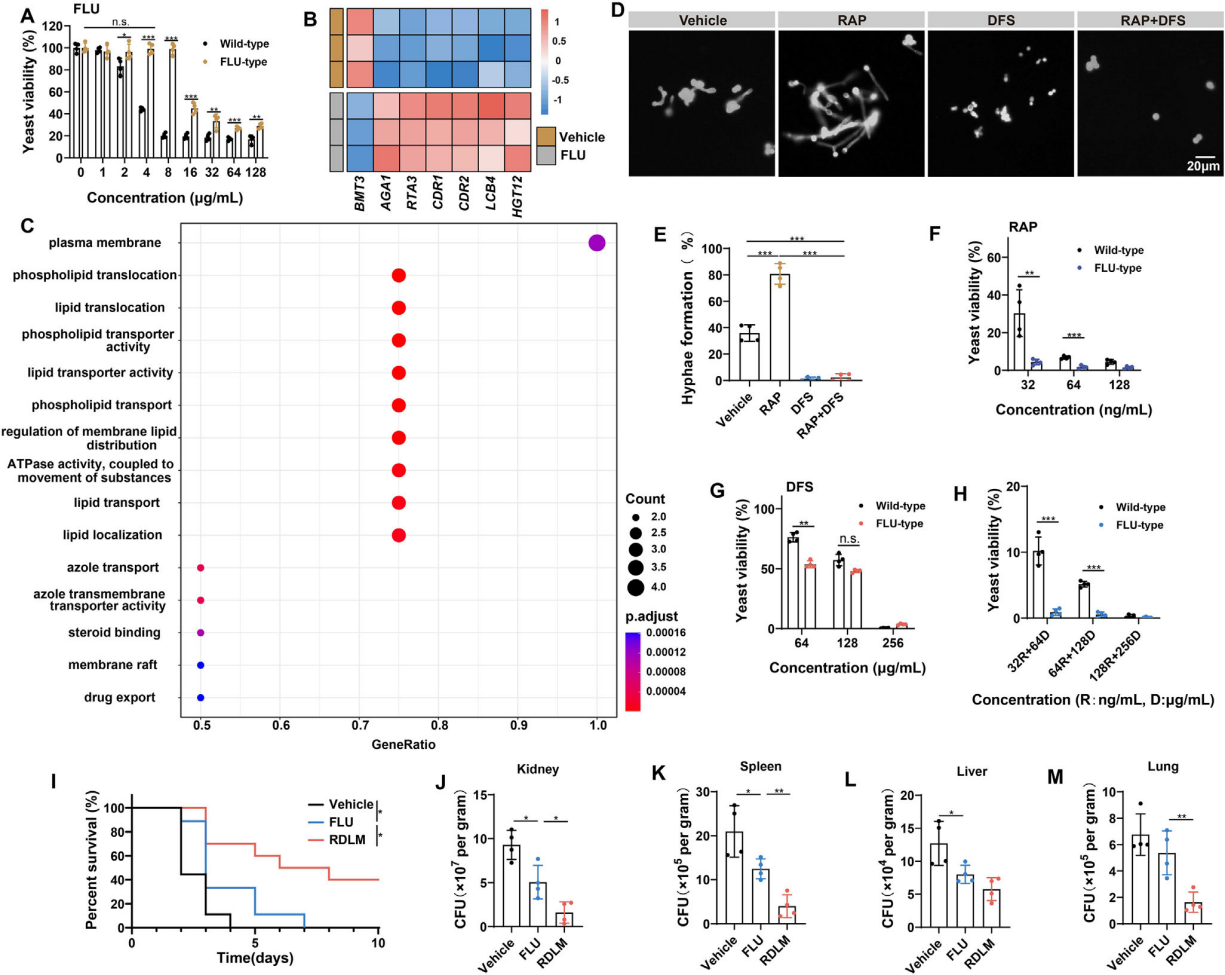
Synthetic lethal therapy for FT *C. albicans*. A) The yeast viability of WT and FT *C. albicans* after FLU treatment of various concentrations (*n* = 4). B) The heat map of the altered genes from WT and FT *C. albicans*. The red squares indicate the increased transcription of related genes (*n* = 3). C) GO pathway analysis of the biological differences between WT and FT *C. albicans*. D) The morphology of FT *C. albicans* in liquid medium after various treatments (scale bar = 20 µm). E) Quantification of hyphae formation rate from (D) (*n* = 4). F–H) The yeast viability of *C. albicans* (WT and FT) after various treatments of various concentrations (*n* = 4), including RAP (F), DFS (G), and RAP+DFS (H). I) Survival curve of model mice infected with FT *C. albicans* ([FLU] = 1 mg kg^−1^, [RAP] = 0.5 mg kg^−1^, [DFS] = 5 mg kg^−1^; *n* = 9,10). J–M) The CFU of FT *C. albicans* in the kidney (J), spleen (K), liver (L), and lung (M). The tissues were harvested from the infected mice at 30 h after different treatments (*n* = 4). Data are shown as mean ± SD; *p < 0.05; **p < 0.01; ***p < 0.001. n.s. indicates non‐significance.

The emergence of fungal drug resistance, particularly to FLU, is escalating into a pressing concern. To date, RAP and DFS have not yet been clinically administered for *C. albicans* infections, but they target mechanisms distinct from traditional antifungal drugs. Therefore, RAP and DFS may exhibit superior therapeutic effects against existing drug‐resistant strains. To mimic the clinical emergence of drug‐resistant fungi, we obtained a fluconazole‐resistant type (FT) *C. albicans* strain by FLU pressure screening. At a concentration of 4 µg mL^−1^, FLU exhibited an inhibitory rate of 47.8% against WT *C. albicans*, but only 6.9% against the FT *C. albicans* (**Figure** **8**A). The transcriptomics analysis revealed that only eight genes of the drug‐resistant strain changed significantly compared with the WT *C. albicans* (Figure S11, Supporting Information). Notably, the transcription levels of *CDR1* and *CDR2* encoding ABC transporters in FT *C. albicans* increased, indicating that FT *C. albicans* could excrete intracellular drugs to the extracellular space, resulting in drug resistance.^[^ ^38^ ^]^ *LCB4* and *RTA3*, which play roles in cell membrane synthesis, were up‐regulated, potentially enhancing the stability of the cell membrane and subsequently leading to increased drug resistance. Conversely, the down‐regulation of *BMT3* impeded the synthesis of β‐1,3‐glucan, which is the target of echinocandins. Consequently, FT *C. albicans* may exhibit cross‐resistance to echinocandins (Figure 8B). GO analysis showed that the functions of azole transport, azole transmembrane transporter activity, drug export, and membrane raft of FT *C. albicans* had changed significantly, indicating that the physiological changes of the drug‐resistant strain were mainly related to drug efflux (Figure 8C). After treatment with FLU, there was no significant change in hyphal growth of FT *C. albicans*, and the hyphal length was similar to that of the control group. This result indicates that FLU is unable to resolve the dimorphism issue of *C. albicans* (Figure S12A,B, Supporting Information). The effects of DFS and RAP on the hyphae of FT *C. albicans* are similar to those on WT *C. albicans*. Specifically, DFS can significantly inhibit the hyphal growth of FT *C. albicans*, while RAP can significantly promote it. When DFS and RAP act in combination, DFS can counteract the hypha‐promoting effect of RAP, thereby maintaining over 97% of FT *C. albicans* in the yeast form (Figure 8D,E). The inhibitory effect of RAP and DFS on the growth of FT *C. albicans* remained unaffected. Notably, they exhibit a superior inhibitory effect on FT *C. albicans* compared to WT *C. albicans* at lower concentrations. This could be because RAP and DFS are not substrates of azole drug transporters, and alterations in the membrane structure of FT *C. albicans* may also improve their cellular entry efficiency. The combination of RAP and DFS exhibited an enhanced therapeutic effect, achieving near‐complete killing (close to 100%) at a concentration of 32 ng mL^−1^ RAP combined with 64 µg mL^−1^ DFS (Figure 8F–H). In a mouse model of systemic infection with FT *C. albicans*, all mice in the FLU group succumbed by the 8th day. In contrast, the survival rate of mice in the RDLM group reached 40% on the 10th day, marking a significant extension in the survival time of mice infected with the FT *C. albicans* (Figure 8I). Compared with FLU, RDLM can significantly reduce the load of *C. albicans* in the kidney, spleen, liver, and lung, which is only 17.2%, 19%, 45.4%, and 24.1% of the control group, respectively (Figure 8J–M; Figure S12C, Supporting Information). Therefore, RDLM has a favorable therapeutic effect on drug‐resistant *C. albicans* systemic infection.

## 3 Discussion

Due to the misuse of antibiotics, drug resistance has given rise to increasingly severe global health issues. In the foreseeable future, the emergence and dissemination of super fungi will pose significant challenges to drug research and development. Numerous reports indicate a worsening trend in Candida's resistance to existing medications, exemplified by innate resistance of *Candida krusei* to FLU and decreased sensitivity of *Candida glabrata* to azoles and echinocandins.^[^ ^39^, ^40^, ^41^ ^]^ Over the past four decades, the progress in developing novel antifungal drugs has been sluggish, with FLU remaining the primary treatment for systemic infections caused by *C. albicans*. Repurposing existing drugs can mitigate research and development costs, expedite the process, and enhance the interest of pharmaceutical companies in this area. RAP, which has been extensively utilized in organ transplantation and cancer treatment with proven safety, acts as an inhibitor of the mTOR pathway, distinguishing it from current anti‐*C. albicans* medications in terms of its target.^[^ ^42^ ^]^ We have also discovered that RAP exhibits a robust anti‐*C. albicans* effect in vitro, achieving an inhibition rate exceeding 90% at a concentration of 64 ng mL^−1^. Notably, drug‐resistant strains of *C. albicans* cannot resist this novel drug (Figure 8H). FT *C. albicans* has a marked elevation in the transcription levels of *CDR1* and *CDR2*, which encode ABC transporters. However, the therapeutic efficacy of RAP remains unaffected, suggesting that RAP may not serve as a substrate for these ABC transporters and can potentially serve as a “ back force” for existing therapeutic agents (Figure 8B).

However, the effectiveness of free RAP in mice with systemic *C. albicans* infection is unsatisfactory, failing to improve the survival rate of the model mice. In antifungal therapy, the body's immune system plays a crucial role, including phagocytosis and clearance of pathogens by macrophages. As an immunosuppressant, RAP inevitably impacts the function of immune cells, thereby failing to demonstrate its antifungal efficacy. Utilizing bionic technology with the specific targeting effect of dectin‐1 can significantly mitigate the adverse impact of RAP on immune cells, enhance drug concentration in infected areas, and achieve synergistic and attenuation effects. After treatment with RAP, the transcription level of the gene involved in β‐glucan synthesis (*LKH1*) in *C. albicans* was upregulated, potentially facilitating the targeting ability of nano‐drugs to *C. albicans* and promoting the interaction between dectin‐1 and β‐glucan (Figure 4E). Furthermore, RAP can induce the transformation of *C. albicans* into the hyphal phase, which may be an adaptive response to nutritional pressure or a key factor contributing to the compromised therapeutic effect of RAP in vivo. Therefore, a comprehensive evaluation of the impact of new drugs on the immune system, biphasic transition, and antifungal activity will be beneficial in improving the success rate of drug research and development in the future. The transcriptomics data and the investigation of the knockout mutant indicate that the transition to the hyphal state may be associated with the upregulation of *HDA1*. In addition, how RAP affects the expression and activity of *HDA1* is worthy of further investigation in our future research. Combining inhibitors of the *HDA1*‐related pathway with RAP holds promise for further enhancing the therapeutic effect in systemic *C. albicans* infections.

The transition between yeast and hyphae is a crucial strategy for *C. albicans* to achieve systemic invasion, facilitating blood dissemination, reproduction, adhesion, and invasion. Additionally, biphasic transformation may serve as a defensive mechanism for *C. albicans* against existing antifungal drugs. Currently, available antifungal agents are primarily effective against yeast‐like fungi, and whether these drugs can induce a transformation to a hyphal state, just like RAP, is a noteworthy topic. Iron, an essential trace metal for microbial growth, participates in microbial DNA synthesis, redox reactions, enzyme catalysis, and other metabolic pathways. Our previous research on *C. albicans* hyphae revealed that iron ions can promote the growth of hyphae. Therefore, combining RAP with iron source limitation to control the formation of the hyphal state of *C. albicans* can achieve synthetic lethal therapy based on targeting dimorphism. Compared to monophasic therapy, this synthetic lethal approach significantly reduces fungal loads in various organs and substantially prolongs the survival time of mice with systemic infections. Similar to RAP, DFS maintains its therapeutic effect against drug‐resistant *C. albicans*. Hence, combining DFS with existing drugs, such as azoles and echinocandins, holds promise for improving the treatment and prognosis of systemic infections through dimorphism‐targeted therapy.

After the treatment of DFS, the classical pathway of controlling hyphae growth has not changed. However, *NCE103*, which functions as a sensor for carbon dioxide, has undergone significant alterations. Notably, carbon dioxide induction is a crucial factor in promoting hyphae growth.^[^ ^43^ ^]^ Currently, we lack an understanding of how iron ions affect *NCE103*, and further detailed research is necessary to elucidate the relationship between iron ions and *NCE103*. It is evident that following DFS treatment, the vacuole structure of *C. albicans* is disrupted, resulting in damage to the number and function of ribosomes, and inhibition of the polarization process of hyphae. These effects are the direct causes of DFS's inhibition of hyphae formation in *C. albicans*. Other trace metal elements, such as copper and zinc, can also contribute to hyphae formation to a certain degree, and the addition of DFS can mitigate this hyphae‐promoting effect (Figure S13, Supporting Information). DFS, an iron‐chelating agent, treats patients requiring long‐term blood transfusions due to iron accumulation conditions such as thalassemia. DFS exhibits a high selectivity for Fe^3+^ and binds to iron with a strong affinity ratio of 2:1, while exhibiting a relatively low affinity for zinc and copper.^[^ ^44^ ^]^ Given that the iron content in blood is ≈65 times higher than zinc and 363 times higher than copper, we suppose that DFS primarily exerts its therapeutic effect through iron chelation in vivo. The distinct contributions of other trace elements will be subject to future research.

## 4 Conclusion

Given the therapeutic challenges posed by the dimorphism of *C. albicans*, innovative synthetic lethal therapies have been developed. RAP inhibits the mTOR signaling pathway and effectively eliminates the yeast *C. albicans*. By restricting iron availability, DFS disrupts vacuole structure, impairs ribosome function, and decreases ribosome number, ultimately inhibiting hyphal formation and polarization. In addition, engineered macrophage membranes with high levels of dectin‐1 can improve the targeting of therapeutic agents and minimize the harmful effects on the immune system. This synthetic lethal therapy targeting the dual‐phase morphology of *C. albicans* prevents the fungus from evading the elimination of drugs and the immune system through morphological transformation, thus significantly improving the prognosis for mice with systemic infections caused by drug‐resistant *C. albicans*.

## 5 Experimental Section

### *C*. *albicans* Strains and Cell Lines

*C. albicans* (ATCC 10 231) was cultivated with YM medium at 30 °C. To induce hyphae formation, *C. albicans* was cultured in RPMI 1640 medium containing FeCl_3_ (200 µm) at 37 °C. The mouse macrophage cells (RAW 264.7, RRID: CVCL_0493) were purchased from Fuheng Biology and cultured in DMEM containing 10% FBS at 37 °C. The cell line was confirmed contamination free. To generate FLU‐resistant type strains, *C. albicans* was maintained in the YM liquid medium with 4 µg mL^−1^ of FLU at 30 °C for 24 h. Then, the grown *C. albicans* was collected and washed with PBS three times. The cell suspension was diluted to an OD600 of 0.01 in YM and added FLU (4 µg mL^−1^). After ten cycles, *C. albicans* was stored at −80 °C.

### Construction of *C. albicans* Gene Deletion Mutants

The knockout of the *HDA1* and *YPT72* genes was performed using a fusion PCR‐mediated homologous recombination strategy. Taking the *had1*Δ strain as an example: using the whole genome of *C. albicans* (ATCC 10 231) as the template, the upstream homologous arm (≈1000 bp) and downstream homologous arm (≈1000 bp) of the target *HDA1* gene in *C. albicans* were amplified with Primer 1/Primer 2 and Primer 5/Primer 6 as primers, respectively; using plasmid pTH10 as the template, the *SAT1* flipper was amplified with Primer 3 and Primer 4 as primers. Using Primer 1 and Primer 6 as primers, the purified upstream and downstream homologous arm fragments of the target *HDA1* gene, together with the selectable marker gene *SAT1*, were fused and amplified via fusion PCR. The knockout cassette was transformed into the WT *C. albicans* to generate the *hda1*: *SAT1*/*HDA1* using the Frozen‐EZ Yeast Transformation II Kit (Zymo Research). Transformants that could grow on YPD agar plates (containing 100 µg mL^−1^ nourseothricin) were validated by colony PCR for the integration of *SAT1*. *Ypt72*Δ mutant was prepared following the same protocol with primer 7–12. All the primers were listed in the supporting information (Table S4, Supporting Information).

### Extraction of Macrophage Membrane

The lentivirus, engineered to overexpress dectin‐1, was transfected into RAW 264.7 cells. These transfected cells were screened with puromycin and indicated by green fluorescence. To isolate the cell membrane, the macrophages were harvested, washed with PBS, and added with hypotonic lysate (1 mm NaHCO_3_, 0.2 mm EDTA, and 1 mm PMSF). Macrophages were lysed at 4 °C and centrifuged for 5 min (3200 g). The supernatant was collected and centrifuged for 20 min (4 °C, 20 000 rpm). The sediment was suspended in PBS to obtain the cell membrane.

### Preparation and Characterization of RDLM

To prepare RDL, 240 mg lecithin was added to 3 mL water. The mixture was heated to 55 °C and stirred to make it uniform. 1 mL of DFS (8 mg mL^−1^, dissolved in ethanol) and 0.5 mL of rapamycin (1.6 mg mL^−1^, dissolved in ethanol) were added. The mixed solution was ultrasonicated for 4 min (300 W) to obtain RDL. The free drug was removed by dialysis for 18 h. RDL and cell membrane solution were evenly mixed and extruded through porous polycarbonate membranes of 400 and 200 nm to form RDL@MM (RDLM). RLM and DLM were prepared similarly. To determine the concentration of RAP and DFS, various nanomedicines were extracted with methyl alcohol (RAP) or methanol (DFS), and sonicated for 10 min. The supernatant was collected and determined by high‐performance liquid chromatography (HPLC, LC40, Shimadzu, Japan) equipped with a C18 column. RAP's mobile phase consisted of 75% (v/v%) acetonitrile and 25% (v/v%) ultrapure water, and DFS's mobile phase consisted of 75% (v/v%) methanol and 25% (v/v%) 0.1% (v/v%) phosphoric acid. The size distributions and *ζ* potentials were determined using the Zetasizer Nano (Malvern Instruments, Malvern, U.K.). The morphology of the nanomedicines was characterized by transmission electron microscopy (TEM, JEM‐2100F, JEOL, Japan).

### Hyphal Formation Assays

To investigate the impact on hyphae formation, the *C. albicans* suspension was diluted to an OD600 of 0.3 in RPMI 1640 medium enriched with 200 µm FeCl_3_. Following the introduction of various drugs into the liquid medium ([RAP] = 32 ng mL^−1^, [DFS] = 256 µg mL^−1^), *C. albicans* was cultured for 6 h at 37 °C. The cells were centrifuged and washed with PBS three times. Before microscope observation (TS‐2, Nikon), calcofluor white (Sigma, 18909‐100ML‐F) was added to stain the cell wall of fungi. To assess the control of tissue invasion in vitro, RAP or DFS was incorporated into 1% agar media before solidification ([RAP] = 32 ng mL^−1^, [DFS] = 256 µg mL^−1^). The *C. albicans* suspension was adjusted to an OD600 of 0.3 with RPMI 1640. Ten microliters of strain suspension was spotted on the solid medium containing RPMI 1640 and 200 µm FeCl_3_. The plates were incubated at 37 °C for 10 days. The top view of the colonies was photographed by the camera. The diameter of the central area of each colony, as well as the distance between the point of hyphal insertion and the edge of this central area, was measured. To explore the penetration of hyphae into agar, the colonies were vertically sectioned through their centers, producing slices of 1 mm thickness. These vertical sections were then photographed under a microscope. The depth of hyphae invasion into the agar was analyzed using Image J software.

### The Analysis of Vacuolar Structure and Yeast Viability

The *C. albicans* suspension was adjusted to an OD600 of 0.3 in YM medium. *C. albicans* was treated with different medicines for 12 h. Then, strains were collected and divided into two parts. One part was incubated with CellTracker Blue CMAC (Thermo Fisher, Y7531, vacuolar dye, 100 µm) for 30 min and washed with PBS three times before imaging. Another one was incubated with FUN 1 probe (Thermo Fisher, F‐7030, 10 µm) for 30 min to assess yeast viability. Images were acquired by using a microscope

### The Assessment of Macrophage Function

RAW 264.7 cells were added to a 24‐well plate covered with a glass plate with a density of 3 × 10^5^ per well. RL and RAP (RAP = 10 µm) were added for 24 h. *C. albicans* was stained with calcofluor white, and adjusted to 3 × 10^6^ CFU mL^−1^. After treatment with different medicines for 12 h, the *C. albicans* was incubated with macrophages for another two hours. Macrophages were washed twice with PBS to remove the free *C. albicans* and stained with DID (KeyGENE, KGE2602‐10) for 20 min. Images were acquired by using a microscope. To investigate the impact of RAP on macrophages, macrophages were pre‐treated with different medicines and then incubated with *C. albicans*.

### Transcriptomics and Bioinformatics Analysis

After undergoing various therapeutic interventions, *C. albicans* samples were collected. Total RNA was extracted and assessed for both quality and quantity through agarose gel electrophoresis, a Nano Photometer spectrophotometer, and a Bioanalyzer 2100 system. Following this, the mRNA was purified and deliberately fragmented using chemical reagents under controlled high temperatures. These fragmented RNA segments served as templates for synthesizing the initial cDNA strand, a process facilitated by random primers. Subsequently, the adapter‐modified fragments underwent purification and amplification to produce the final cDNA library, which was then quantified using Pico Green dye and a fluorescence spectrophotometer. Sequencing of the cDNA was performed on the Illumina HiSeq platform, utilizing synthesis technology. The resulting paired‐end clean reads were aligned to the reference genome with Hisat2 v2.0.5, and Stringtie was employed to count the reads attributed to each gene. Finally, the FPKM (Fragments Per Kilobase of transcript per Million mapped fragments) value for each gene was computed. Differential expression analysis was carried out using the DESeq2 R package, where *P*‐values were adjusted using the Benjamini and Hochberg method to maintain control over the false discovery rate. Genes exhibiting an adjusted *P*‐value of less than 0.05 were classified as differentially expressed. For these genes, Gene Ontology (GO) enrichment analysis was conducted using the clusterProfiler R package. Furthermore, Kyoto Encyclopedia of Genes and Genomes (KEGG) enrichment analysis was performed to identify the primary categories of the differentially expressed genes (DEGs), leveraging molecular‐level information.

### Evaluation of Therapeutic Efficiency In Vivo

All animals were obtained from SLAC Laboratory Animal Company (Shanghai, China) and handled under the supervision of the Animal Care and Use Committee (IACUC) of Nanjing Normal University. In the systemic infection model, C57BL/6 mice (6–8 weeks) were intravenously injected with *C. albicans* yeast suspended with PBS (3 × 10^7^ CFU mL^−1^, 100 µL), and randomly grouped. A dose of 5 × 10^7^ CFU mL^−1^, 100 µL) was for the severe infection model. Different formulations were injected 6 h post‐infection with *C. albicans* (RAP = 0.5 mg kg^−1^, DFS = 5 mg kg^−1^). Lifetime was recorded within ten days. In a separate experiment, model mice were euthanized 30 h after different treatments. The liver, spleen, lung, and kidney were weighed and ground with sterile PBS. After a suitable dilution, the grinding solution was coated on the solid medium of Candida coloration at 30 °C for 48 h. The number of *C. albicans* colonies on the plate was counted. In another independent experiment, according to the above experimental steps, the organs for H&E staining and hexamine silver staining were harvested. To determine the biodistribution of RDLM, infected mice were treated with IR775‐labeled RDLM or free IR775 by intravenous injection. Fluorescence images were acquired using an IVIS Lumina imaging system (Ex = 745 nm, Em = 820 nm, Xenogen Corporation‐Caliper, Alameda, CA, U.S.A.).

### Statistical Analysis

Statistical analysis was performed via GraphPad Prism 8 software. Data were reported as mean ± SD. Statistical significance was ascertained by a two‐tailed Student's *t‐*test for two groups and a one‐way analysis ANOVA for multiple groups. A value of *p* < 0.05 was considered statistically significant.

## Conflict of Interest

The authors declare no conflict of interest.

## Author Contributions

Y.G., J.S., and J.Y. contributed equally. H.H., Y.L., L.W., and L.M. conceived the project and designed the experiments. Y.G., J.S., and J.Y. performed the experiments. G.Y. and J.Y. analyzed the data. Q.C., Y.W., Y.X., and R.X. helped to collect some of the data. This manuscript was written through the contributions of all authors. All authors approved the final manuscript.

## Supporting information



Supporting Information

## Data Availability

The data supporting the results of this study are available within the paper and its Supplementary Information. The raw and processed data generated in this study have been deposited in the database under accession codes PRJNA1175751 (transcriptomics data).
