# Supplementary material for: Synthetic Lethal Therapy Based on Dimorphism for Systemic Infection of Drug‐Resistant Candida albicans
Source: Adv Sci (Weinh). 2025 Dec 3;13(11):e18196. doi: 10.1002/advs.202518196 (PMC12931174; doi:10.1002/advs.202518196)
Supplement: Supplementary file 1 — Supporting Information [file ADVS-13-e18196-s001.docx]

Supporting information

**Synthetic Lethal Therapy Based on Dimorphism for Systemic Infection of Drug-resistant *Candida albicans***

***Yang Gao^1,#^, Jiahe Su^1,#^, Jiawei Yuan^1,#^, Qinyan Cao^1^, Yue Wu^1^, Yuyang Xiao^1^, Guang Yang^1,2^,*** ***Ruomu Xia^3^, Jingpeng Yang^1^, Yanan Li^1,^*, Lina Wu^1,^*, He Huang^1,^*, Lingtong Meng^1,^****

**Affiliations:**

1. State Key Laboratory of Microbial Technology, School of Food and Pharmaceutical Engineering, Nanjing Normal University, Nanjing 210023, China.
2. Shanghai Institute for Advanced Immunochemical Studies, ShanghaiTech University, Shanghai 201210, China.
3. College of life science, Beijing Normal University, Beijing 100091, China.

# These authors contributed equally.

***Author for correspondence:**

Lingtong Meng: mlt@njnu.edu.cn

He Huang: huangh@njnu.edu.cn

Lina Wu: wuln@njnu.edu.cn

Yanan Li: liyanan@njnu.edu.cn


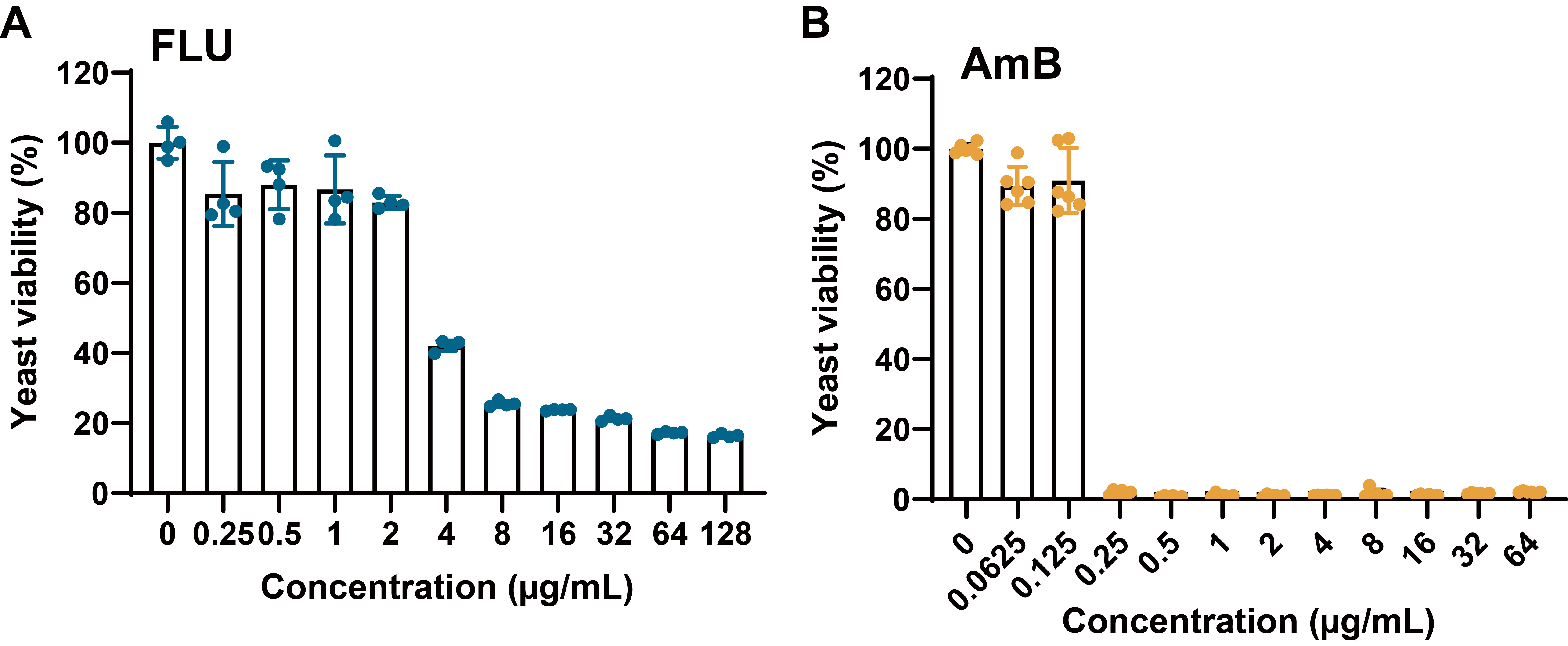


**Figure S1.** Inhibitory effect of fluconazole (FLU) and amphotericin B (AmB) on yeast. The yeast viability of *Candida albicans* (*C. albicans*) after fluconazole (A) or amphotericin B (B) treatment of various concentrations (n=4 and 6). Data are shown as mean ± SD.


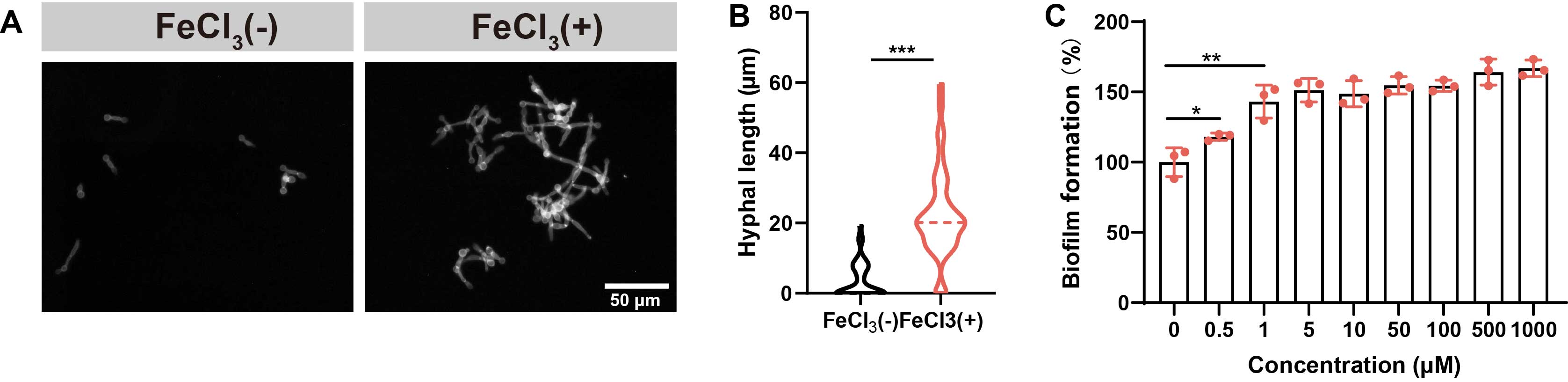


**Figure S2.** (A-B) The hyphal morphology of *C. albicans* strains stained with calcofluor white in liquid medium supplemented with FeCl_3_ (scale bars=50 μm) and quantification of hyphal length from (A), the horizontal line represents the median vale (n=40). (C) Biofilm formation after the addition of FeCl_3_ of different concentrations. The biofilm content was determined by crystal violet staining (n=3). Data are shown as mean ± SD. *p<0.05; **p<0.01; ***p<0.001.


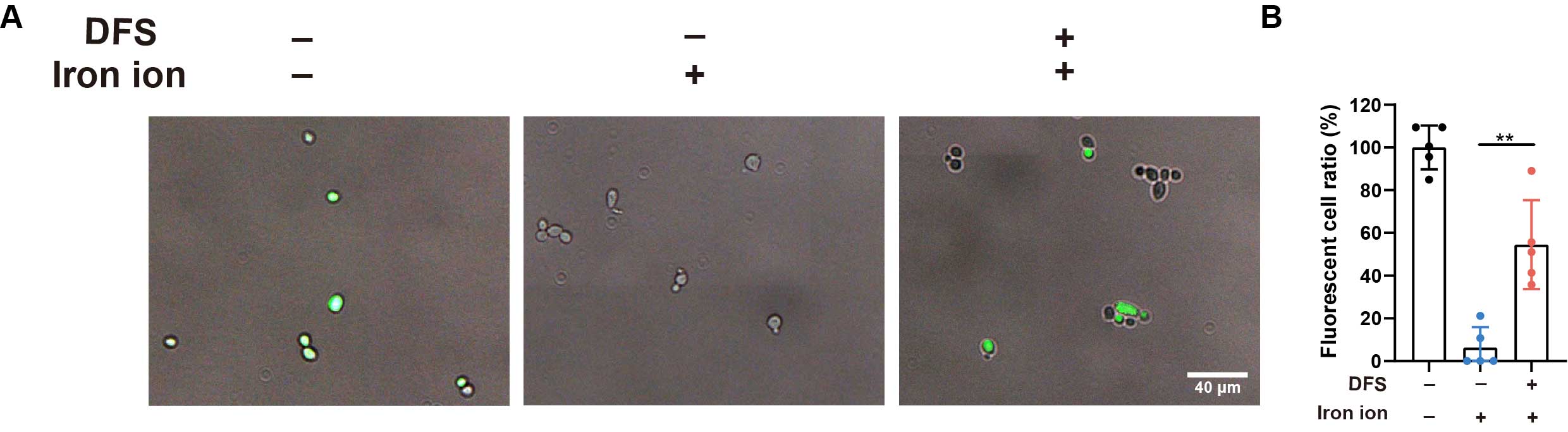


**Figure S3.** Iron ion content in yeast cells after various treatments. (A-B) The images of *C. albicans* stained by calcein-AM after exposure to iron ions (200 μM) and DFS (256 μg/mL) for 1 h and the quantification of fluorescent cell ratio. (scale bars=40 μm, n=5). Upon entering normal cells, calcein-AM emits green fluorescence. However, high concentrations of iron ions within the cells cause this fluorescence to quench. Data are shown as mean ± SD; **p < 0.01.


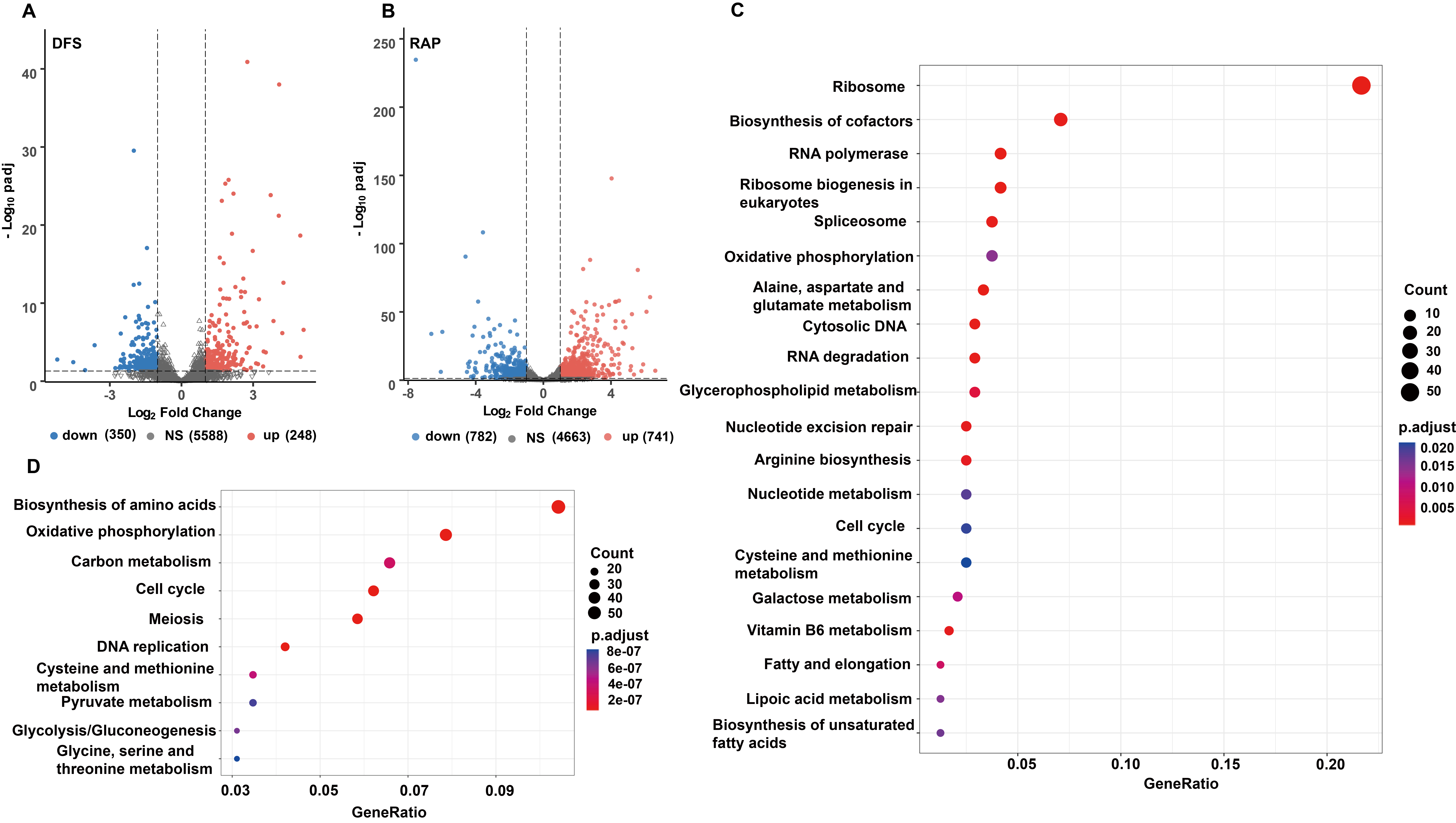


**Figure S4.** The transcriptomics analysis of *C. albicans* after rapamycin (RAP) and deferasirox (DFS) treatment. (A-B) The volcano map shows the changes of genes in *C. albicans* after DFS(A) or RAP(B) treatment. (C-D) KEGG analysis of *C. albicans* after DFS (C) or RAP (D) treatment.

**Table S1.** Altered genes encoding ribosomal 60S subunit protein.

| Gene | log_2_ Fold Change | Description |
| --- | --- | --- |
| *RPL30* | -1.410 | ribosomal 60S subunit protein L30 |
| *RPL37B* | -1.141 | ribosomal 60S subunit protein L18A |
| *RPL3* | -1.228 | ribosomal 60S subunit protein L3 |
| *RPL42* | -1.738 | ribosomal 60S subunit protein L42A |
| *RPL12* | -1.468 | ribosomal 60S subunit protein L12A |
| *RPL29* | -1.511 | ribosomal 60S subunit protein L29 |
| *RPL9B* | -1.472 | ribosomal 60S subunit protein L9B |
| *RPL35* | -1.882 | ribosomal 60S subunit protein L35A |
| *RPL28* | -1.576 | ribosomal 60S subunit protein L28 |
| *RPL23A* | -1.819 | ribosomal 60S subunit protein L23B |
| *RPL13* | -1.332 | ribosomal 60S subunit protein L13A |
| *RPL14* | -1.316 | ribosomal 60S subunit protein L14B |
| *RPL16A* | -1.027 | ribosomal 60S subunit protein L16A |
| *RPL39* | -1.515 | ribosomal 60S subunit protein L36A |
| *RPL10A* | -1.046 | ribosomal 60S subunit protein L1A |
| *RPL27A* | -1.491 | ribosomal 60S subunit protein L27A |
| *RPL38* | -1.377 | ribosomal 60S subunit protein L38 |
| *RPL32* | -1.269 | ribosomal 60S subunit protein L32 |
| *RPL24A* | -1.318 | ribosomal 60S subunit protein L24A |
| *RPL11* | -1.410 | ribosomal 60S subunit protein L11B |
| *RPL19A* | -1.334 | ribosomal 60S subunit protein L19A |
| *RPL10* | -1.101 | ribosomal 60S subunit protein L10 |
| *RPL40B* | -1.212 | ribosomal 60S subunit protein L40B |
| *RPL15A* | -1.011 | ribosomal 60S subunit protein L15B |
| *RPL21A* | -1.093 | ribosomal 60S subunit protein L21A |

**Table S2.** Altered genes encoding ribosomal 40S subunit protein.

| Gene | log_2_ Fold Change | Description |
| --- | --- | --- |
| *RPS6A* | -1.239 | ribosomal 40S subunit protein S6A |
| *RPS9B* | -1.150 | ribosomal 40S subunit protein S9B |
| *RPS13* | -1.258 | ribosomal 40S subunit protein S13 |
| *RPS24* | -1.344 | ribosomal 40S subunit protein S24B |
| *RPS18* | -1.350 | ribosomal 40S subunit protein S18B |
| *RPS28B* | -1.156 | ribosomal 40S subunit protein S28B |
| *RPS10* | -1.135 | ribosomal 40S subunit protein S10A |
| *RPS23A* | -1.678 | ribosomal 40S subunit protein S23B |
| *RPS21B* | -1.609 | ribosomal 40S subunit protein S21B |
| *RPS25B* | -1.162 | ribosomal 40S subunit protein S25B |
| *RPS26A* | -1.433 | ribosomal 40S subunit protein S26A |
| *RPS22A* | -1.132 | ribosomal 40S subunit protein S22A |
| *RPS19A* | -1.182 | ribosomal 40S subunit protein S19A |
| *RPS42* | -1.057 | ribosomal 40S subunit protein S4A |
| *RPS3* | -1.378 | ribosomal 40S subunit protein S3 |
| *RPS14B* | -1.175 | ribosomal 40S subunit protein S14B |


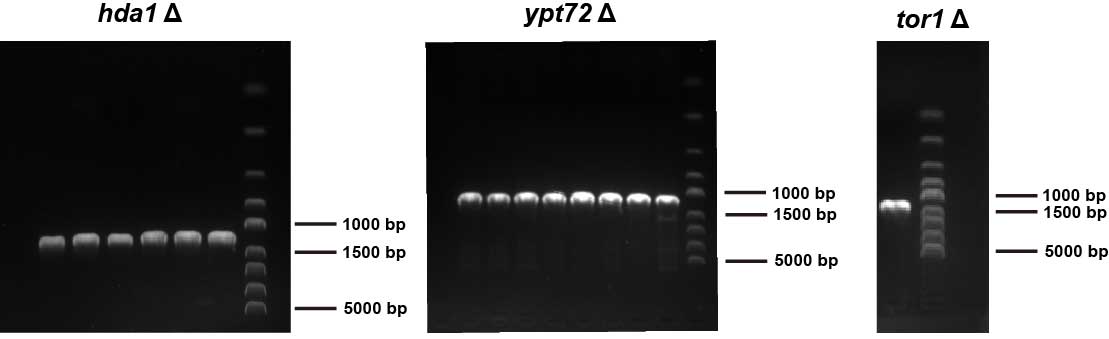


**Figure S5.** PCR identification of *SAT1* gene (1142 bp) from *hda1*Δ mutant, *ypt72*Δ mutant and *tor1*Δ mutant. The presence of the *SAT1* gene indicates that the target gene (*HDA1*, *YPT72* or *TOR1*) has been knocked out.


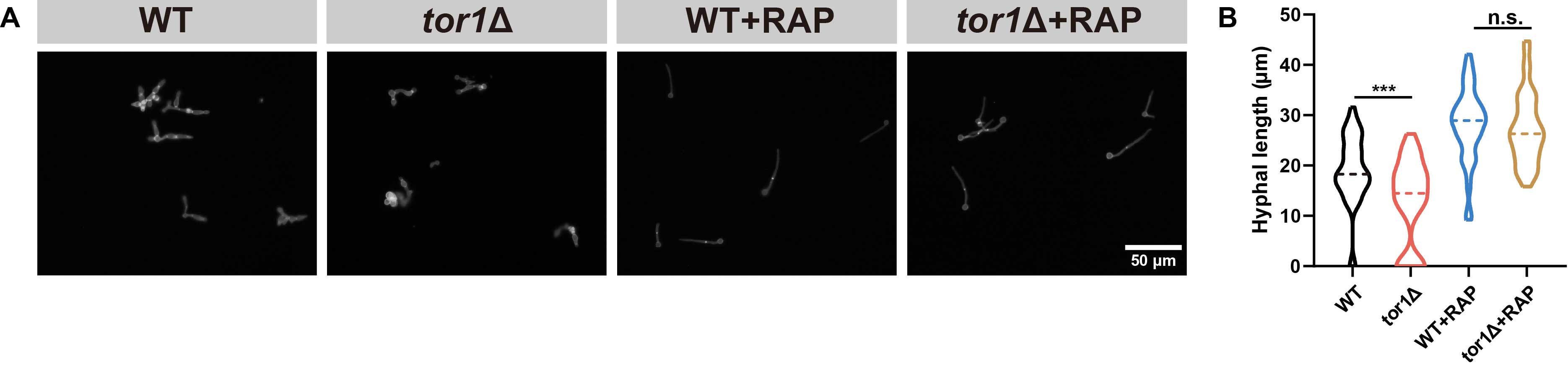


**Figure S6.** Effect of RAP on the hyphal development of *tor1*Δ mutant. (A) The morphology of different *C. albicans* strains (WT and *tor1*Δ) in liquid medium after various treatments (scale bar=50 μm). (B) Quantification of hyphal length from (A), the horizontal line represents the median vale (n=40). ***p < 0.001. n.s. indicates non-significance.

**Table S3.** Genes in the classical pathway of hyphae growth.

| Gene | P value | log_2_ Fold Change |
| --- | --- | --- |
| *Efg1* | 0.05792 | 1.01181 |
| *Cph1* | 0.07248 | 0.81394 |
| *Mep2* | 0.86756 | -0.07490 |
| *Tup1* | 0.61952 | 0.16809 |
| *Rfg1* | 0.40385 | 0.46345 |
| *Rbf1* | 0.23072 | 0.48193 |
| *Tpk1* | 0.60133 | 0.16454 |
| *Tpk2* | 0.96750 | -0.00712 |
| *Dck1* | 0.84808 | 0.12442 |
| *Cyr1* | 0.11155 | 0.47465 |
| *Gpr1* | 0.07769 | 0.66075 |
| *Rim21* | 0.54932 | 0.14276 |
| *Ngt1* | 0.15841 | 0.34700 |
| *Cdc24* | 0.17162 | 0.29267 |


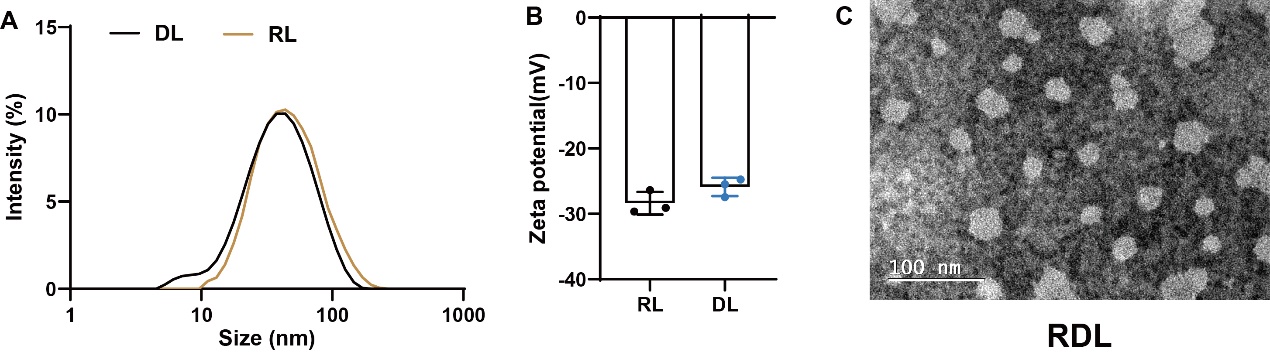


**Figure S7.** (A-B) Size distribution, zeta potential of RL and DL (n=3). (C) Transmission electron microscope image of RDL **(**scale bar=100 nm).


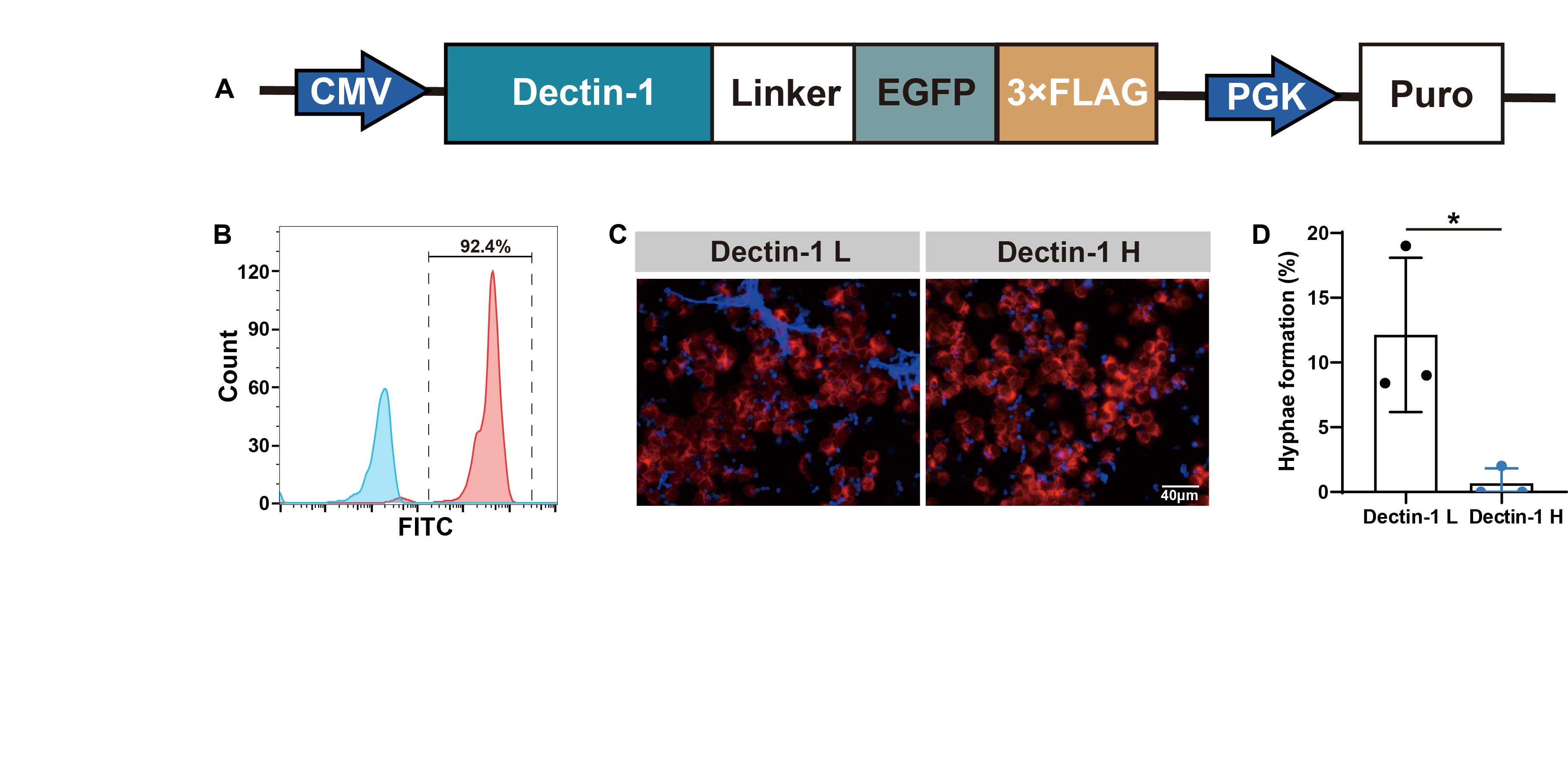


**Figure S8.** Construction and function of macrophages with high expression of dectin-1. (A) Schematic construction of plasmid expressing dectin-1. (B) Flow cytometry of resting macrophage (blue) and high expression of dectin-1 macrophage (red). (C-D) Images of macrophages incubated with *C. albicans* for 2 hours and quantification of hyphae formation (scale bar=40 μm, n=3). L, Low expression of dectin-1; H, High expression of dectin-1. The macrophages were indicated with DiD (red) and *C. albicans* was stained with calcofluor white (blue). Data are shown as mean ± SD; *p<0.05.


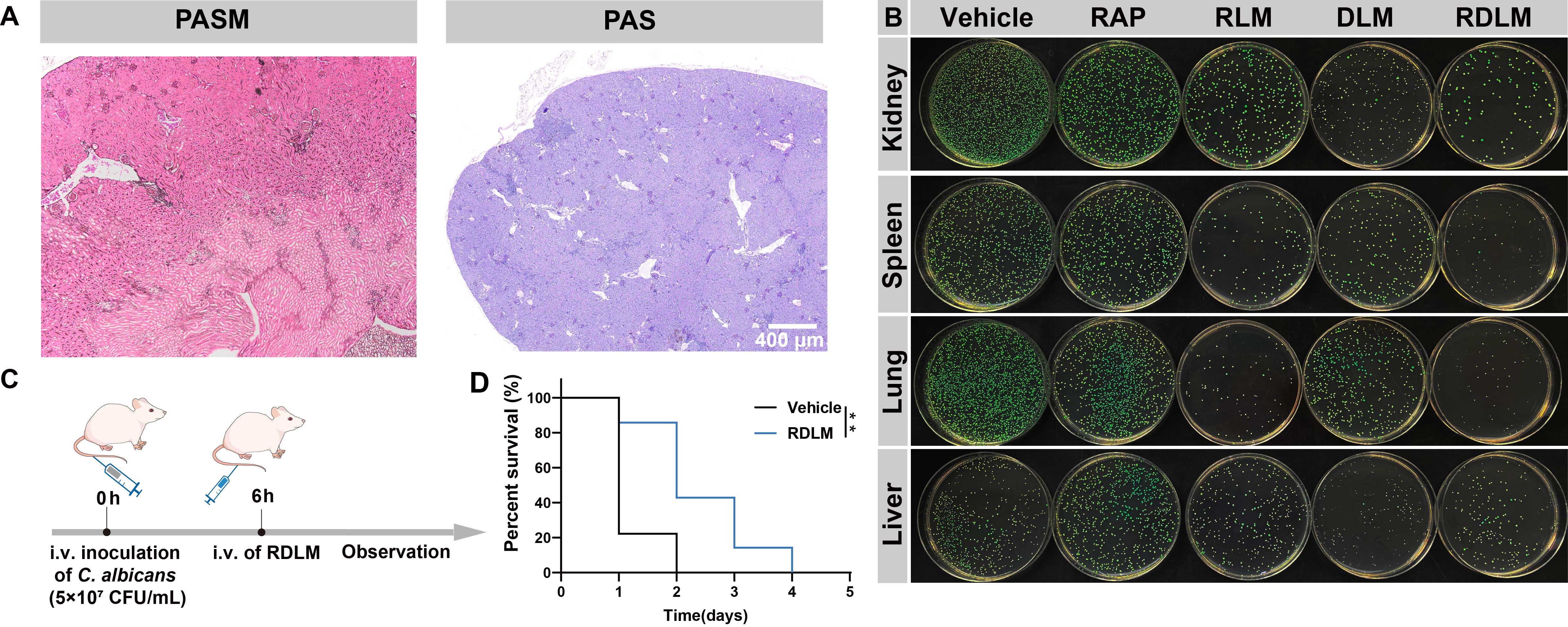


**Figure S9.** Therapeutic effect of infected mice. (A) Representative periodic acid-silver methenamine (PASM) staining and periodic acid-schiff (PAS) staining images of kidney tissue from infected mice after 14 days’ RDLM treatment (scale bar=400 μm). (B) The images of *C. albicans* in *the* kidney, spleen, lung and liver. The tissues were harvested from the infected tissues of mice at 30 h after the different treatments. The green points indicate *C. albicans* colonies. (C) Experimental procedures for survival test in the *C. albicans* severe systemic infection model. (D) Survival curve of severe systemic infection mice after RDLM treatment ([RAP]=0.5 mg/kg, [DFS]=5 mg/kg, n=7-9). Data are shown as mean ± SD; **p<0.01.


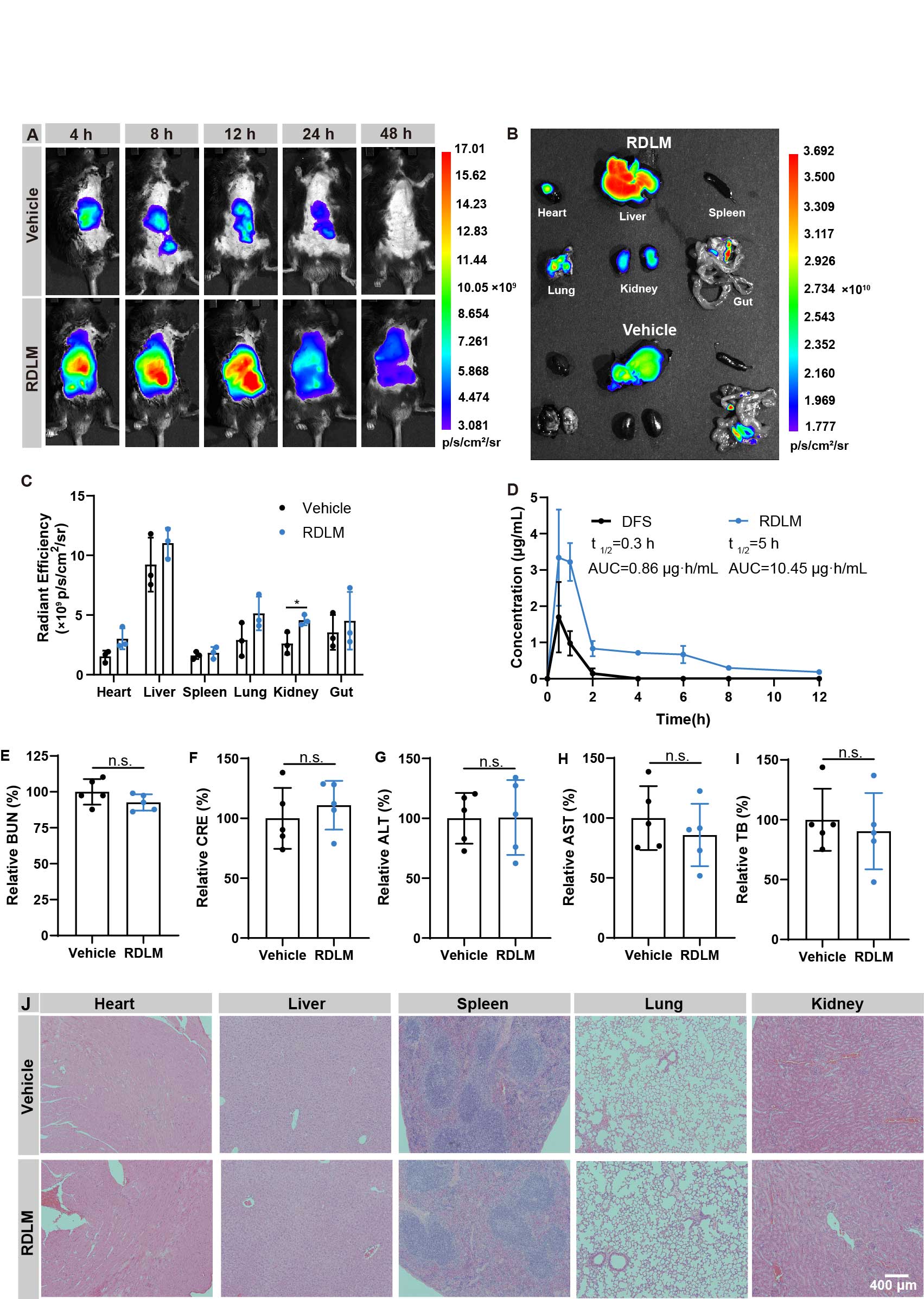


**Figure S10.** Biodistribution and safety evaluation of RDLM. (A-B) Dynamic fluorescence imaging of infected mice and fluorescent image of major organs after intravenous injection of free IR775 and IR775 labeled RDLM. (C) Quantification of average radiant efficiency of major organs(n=3). (D) Plasma drug concentration-time curves of DFS and RDLM (n=3). (E-F) Serum biochemistry data reflecting kidney function including blood urea nitrogen (BUN) and serum creatinine (CRE) (n=5). (G-I) Serum biochemistry data reflecting liver function including alanine aminotransferase (ALT), aspartate aminotransferase (AST), and total bilirubin (TB) (n=5). (J) Representative H&E staining images of tissue sections from mice treated with PBS or RDLM (scale bar=400 μm). Data are shown as mean ± SD; *p < 0.01; n.s. represented non-significance.


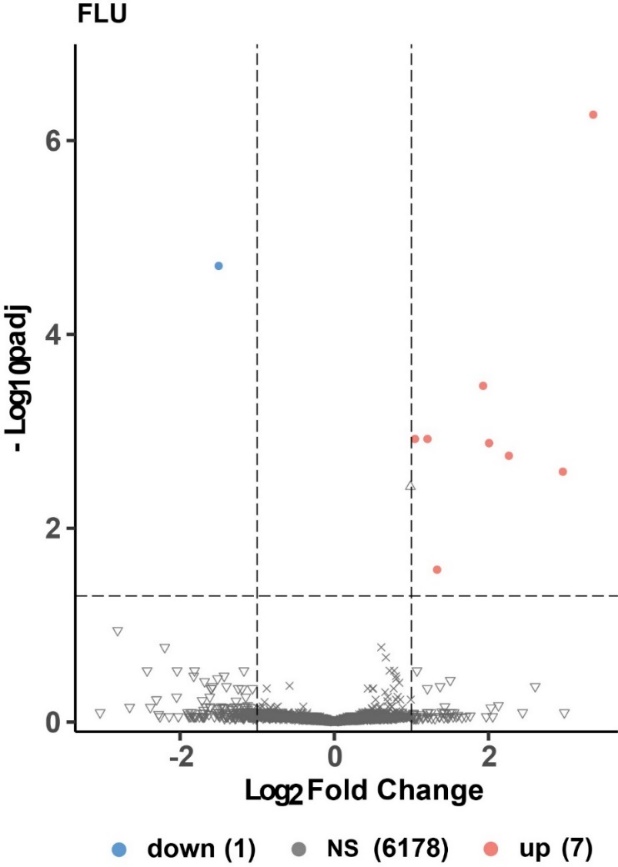


**Figure S11.** The volcano map shows the changes in genes in the fluconazole-resistant type (FT) *C. albicans*.


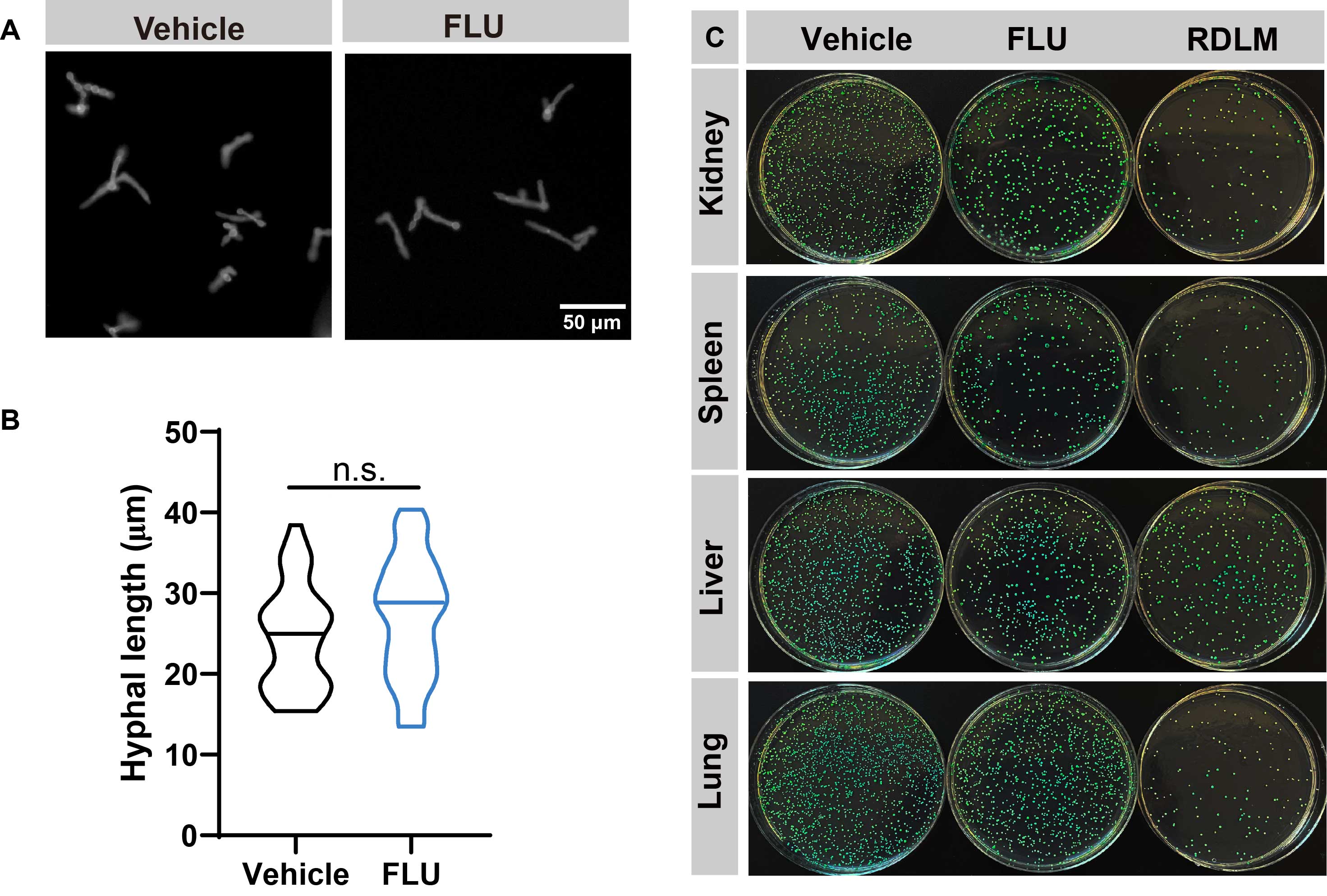


**Figure S12.** (A) The morphology of FT *C. albicans* in liquid medium after different treatments (scale bar=50 μm). (B) Quantification of hyphal length from (A) (n=40). (C) The images of FT *C. albicans* in the kidney, spleen, lung and liver. The tissues were harvested from the infected tissues of mice at 30 h after the different treatments. The green points indicate *C. albicans* colonies. Data are shown as mean ± SD; n.s. indicates non-significance.





**Figure S13.** Effects of metal ions and DFS on hyphae growth. The images of morphological changes in *C. albicans* after exposure to copper ions, zinc ions, iron ions (200 μM), and DFS (256 μg/mL) (scale bar=50 μm). *C. albicans* was cultivated at 37 °C in RPMI 1640 medium, with simultaneous addition of metal ions and DFS to the liquid culture medium. Subsequently, *C. albicans* in each group was stained with calcofluor white to capture morphological images.

**Table S4.** All primers used in this study.

| Name | Sequence (5' to 3') |
| --- | --- |
| Primer1 | GGAAAACCAAAACCTTATCCTG |
| Primer2 | GCCTCCTGTCGCGGTGATTAGTATCGGCCA |
| Primer3 | ACTAATCACCGCGACATGGAGGCCCAGAATACC |
| Primer4 | GCATACCAGAAGCAGTATAGCGACCAGCATTCAC |
| Primer5 | CTGGTCGCTATACTGCTTCTGGTATGCACGACGGT |
| Primer6 | CTAGATGCTGACTTGTTACCTCC |
| Primer7 | GCGCAGTATCAATGTGTCTATACCCG |
| Primer8 | GGCCTCCATGTCCTCTGTATCGTTC |
| Primer9 | AACGATACAGAGGACATGGAGGCCCA |
| Primer10 | CTTCACCTGTGCAGTATAGCGACCAGCATTC |
| Primer11 | TCGCTATACTGCACAGGTGAAGAAAGAGAAC |
| primer12 | GGAAAACCAAAACCTTATCCTG |
